# Next-generation nanomaterials: advancing ocular anti-inflammatory drug therapy

**DOI:** 10.1186/s12951-023-01974-4

**Published:** 2023-08-19

**Authors:** Jing Wei, Jinyu Mu, Yong Tang, Dalian Qin, Junguo Duan, Anguo Wu

**Affiliations:** 1 School of Ophthalmology, Chengdu University of Traditional Chinese Medicine, Chengdu, 610075 China; 2 Present Address: Sichuan Key Medical Laboratory of New Drug Discovery and Druggability Evaluation, Education Ministry Key Laboratory of Medical Electrophysiology, School of Pharmacy, Southwest Medical University, Luzhou, 646000 China

**Keywords:** Ophthalmic inflammatory diseases, Nanomaterials, Biocompatibility, Bioavailability, Controlled release, Targeted delivery, Toxicity

## Abstract

Ophthalmic inflammatory diseases, including conjunctivitis, keratitis, uveitis, scleritis, and related conditions, pose considerable challenges to effective management and treatment. This review article investigates the potential of advanced nanomaterials in revolutionizing ocular anti-inflammatory drug interventions. By conducting an exhaustive analysis of recent advancements and assessing the potential benefits and limitations, this review aims to identify promising avenues for future research and clinical applications. The review commences with a detailed exploration of various nanomaterial categories, such as liposomes, dendrimers, nanoparticles (NPs), and hydrogels, emphasizing their unique properties and capabilities for accurate drug delivery. Subsequently, we explore the etiology and pathophysiology of ophthalmic inflammatory disorders, highlighting the urgent necessity for innovative therapeutic strategies and examining recent preclinical and clinical investigations employing nanomaterial-based drug delivery systems. We discuss the advantages of these cutting-edge systems, such as biocompatibility, bioavailability, controlled release, and targeted delivery, alongside potential challenges, which encompass immunogenicity, toxicity, and regulatory hurdles. Furthermore, we emphasize the significance of interdisciplinary collaborations among material scientists, pharmacologists, and clinicians in expediting the translation of these breakthroughs from laboratory environments to clinical practice. In summary, this review accentuates the remarkable potential of advanced nanomaterials in redefining ocular anti-inflammatory drug therapy. We fervently support continued research and development in this rapidly evolving field to overcome existing barriers and improve patient outcomes for ophthalmic inflammatory disorders.

**Graphical Abstract:**

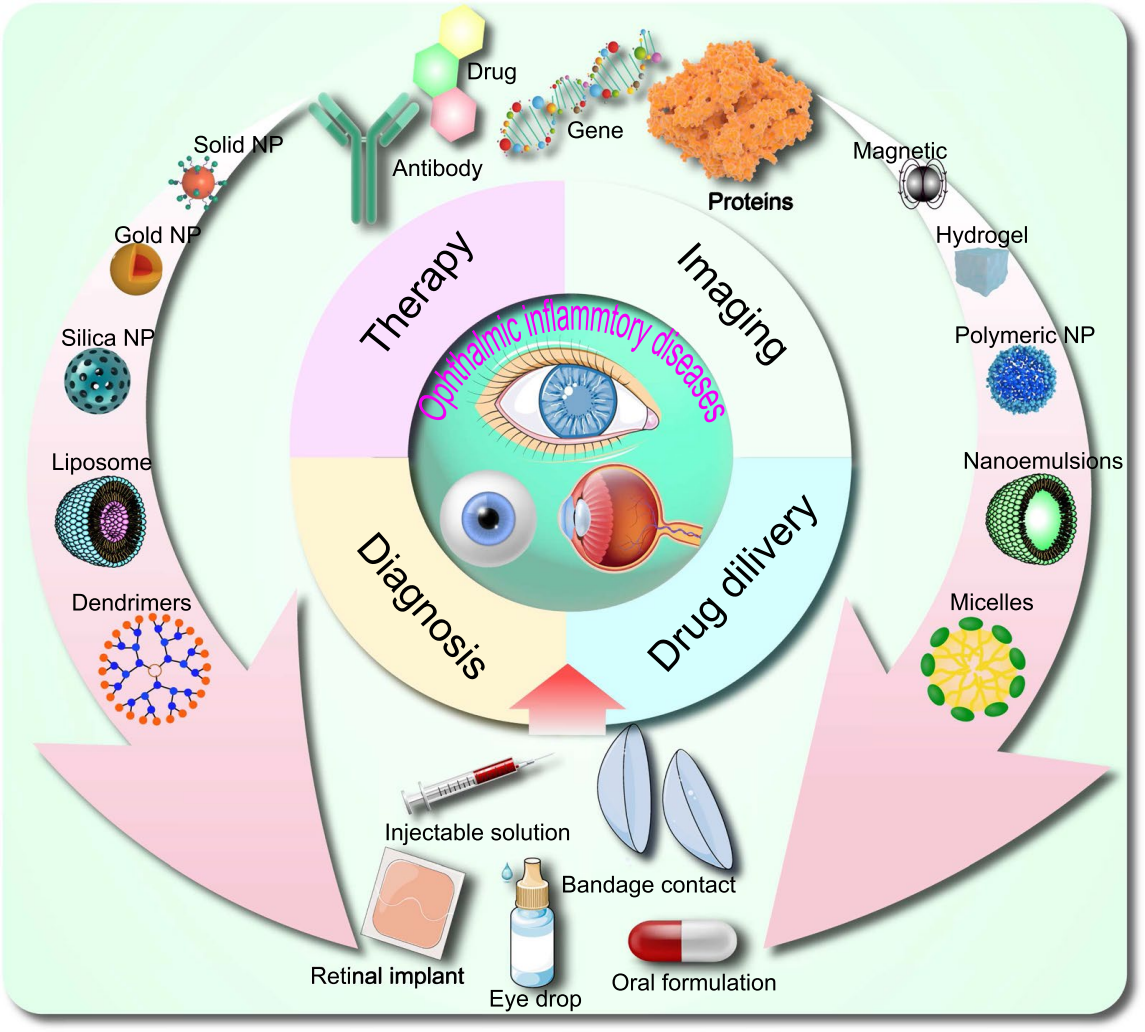

## Introduction

Inflammation-associated ophthalmic diseases comprise a diverse array of ocular disorders characterized by inflammation impacting various eye structures, such as the uvea, sclera, optic nerve, cornea, and retina [1]. The prevalence of these disorders differs considerably, depending on factors like geographic location, population demographics, and the specific condition under consideration. For example, uveitis, a more prevalent inflammatory eye disorder, has an estimated prevalence of approximately 38–730 cases per 100,000 individuals, with higher rates observed in developing countries and certain populations [2]. Conversely, scleritis is less common, with an estimated prevalence of around 4–20 cases per 100,000 people [3]. Optic neuritis (ON) also occurs relatively infrequently, with an estimated prevalence of 1–5 cases per 100,000 individuals, but is more common in populations with a higher incidence of multiple sclero [4]. These conditions can result from various factors, such as infections, genetic predispositions, autoimmune diseases, environmental triggers, or other underlying causes. Typical examples of these disorders include uveitis, scleritis, ON, keratitis, and retinitis, each with unique clinical manifestations and varying severity levels. Symptoms associated with inflammation-related ophthalmic diseases often encompass redness, pain, light sensitivity, blurred or reduced vision, floaters, and, in some instances, sudden vision loss. Diagnosing and managing these conditions necessitate a comprehensive assessment by an ophthalmologist, who may employ a combination of clinical examination, laboratory tests, and imaging studies to ascertain the underlying cause and inflammation severity. Treatment strategies for ocular diseases vary depending on the specific disease and its underlying cause, often involving a combination of approaches. These approaches may include the use of topical or systemic anti-inflammatory medications, such as corticosteroids [5, 6] and nonsteroidal anti-inflammatory drugs (NSAIDs) [6], immunosuppressive therapy with agents like methotrexate or cyclosporine (CsA) [7], administration of antiviral or antibacterial agents in cases of infectious causes [8], and in certain instances, surgical intervention to address complications or unresponsive cases. Prompt detection and appropriate management are crucial to minimize the risk of complications and preserve vision. 

Nanomaterials, defined as materials with at least one dimension ranging from 1 to 100 nm in the nanometer scale [9, 10], exhibit unique physical, chemical, and mechanical properties that markedly differ from those of their bulk counterparts. Owing to their high surface area-to-volume ratio, quantum size effects, and other nanoscale phenomena, they hold promise for advancing the diagnosis and treatment of inflammation-related ocular disorders [11]. For example, NPs, liposomes, and dendrimers can be employed to deliver anti-inflammatory, immunosuppressive, or anti-angiogenic drugs with enhanced targeting, reduced systemic side effects, and sustained release profiles [12]. Quantum dots and gold nanoparticles (AuNPs) can also be utilized in advanced imaging techniques like optical coherence tomography (OCT), fluorescence imaging, and photoacoustic imaging for superior visualization of ocular structures and inflammation for early detection and diagnosis [13]. Quantum dots and AuNPs can also be employed in advanced imaging techniques such as OCT, fluorescence imaging, and photoacoustic imaging to provide better visualization of ocular structures and inflammation for early detection and diagnosis [14, 15]. Additionally, nanofibers, hydrogels, and nanocomposites can function as scaffolds or supports for the regeneration of damaged ocular tissues such as the cornea or retina [16–18]. Continued research in this domain may ultimately result in more effective treatments for inflammation-related eye disorders, with fewer side effects. Despite the extensive information available on nanomaterial formulation, characterization, ocular administration, and targeting, addressing the toxicity and safety of these materials remains an urgent requirement. Consequently, new breakthroughs are essential for facilitating the development and application of next-generation nanomaterials in ocular anti-inflammatory drug therapy.

## Nanomaterials

Nanoparticles (NPs) represent a class of minuscule, synthetically engineered particles with dimensions ranging from 1 to 100 nm. These particles bridge the gap between bulk matter and atoms or molecules. Due to their diminutive size, they exhibit unique characteristics, such as a vast surface area, potent penetrative ability, and stability. NPs find widespread use in diverse fields, including biomedicine, fine chemical engineering, seawater purification, aerospace, environmental energy, and microelectronics. Within the realm of biomedicine, NPs can permeate cellular structures in the body, traverse neural pathways, lymphatic systems, and blood vessels, and selectively accumulate within various cellular architectures. This versatility renders nanoparticle-based materials extensively and actively employed for drug delivery and disease treatment.

Nanomaterials can be primarily classified into two categories: organic and inorganic nanomaterials [19]. Organic nanomaterials encompass polysaccharide-based materials, lipid-based nanomaterials, and polymer-based nanomaterials, such as microspheres, micelles, hydrogels, NPs, dendrimeric macromolecules and nanofibers [20]. Inorganic nanomaterials include magnetic-based materials, gold-based materials, iron oxide-based materials, silica-based materials, and graphene among others [21]. In ophthalmology, nanomaterials have demonstrated promising applications in the diagnosis and treatment of various eye diseases, possessing the capacity to facilitate targeted drug delivery [22], enhance diagnostic imaging [23], and promote tissue regeneration [24]. Nonetheless, further research is required to comprehensively understand the safety and efficacy of these materials in the eye.

### Carbohydrate-based nanomaterials

Carbohydrate-based nanomaterials constitute a class of nanomaterials derived from natural carbohydrates such as cyclodextrins, cellulose and chitosan (CS) [25]. These materials exhibit exceptional physicochemical properties, rendering them highly desirable for a wide array of applications in fields such as medicine, energy and environmental remediation. Carbohydrate-based nanomaterials can be engineered to possess specific properties, including size, shape and surface charge, making them remarkably versatile and suitable for numerous applications. These materials can self-assemble and form complex structures, which are attractive for applications in drug delivery and tissue engineering [26]. Furthermore, carbohydrate-based nanomaterials demonstrate excellent biocompatibility, biodegradability and low toxicity, making them ideal candidates for biomedical applications [27].

#### Chitosan

CS is a linear polysaccharide comprising randomly distributed β-(1–4)-linked D-glucosamine and N-acetyl-D-glucosamine units, originating from partially deacetylated chitin. Due to the presence of protonated amino groups carrying a positive charge, CS exhibits pH-regulating properties and functions as a water-soluble cationic polyelectrolyte capable of interacting with negatively charged molecules. By encapsulating dexamethasone (DEX) sodium phosphate for topical ocular delivery, CS NPs decreased drug residence time in the cornea and enhanced drug permeability [28]. Thus, CS NPs as nano-carriers for DEX have demonstrated broad prospects in the treatment of ocular inflammation.

#### Hyaluronic acid

Hyaluronic acid (HA) is a natural polysaccharide composed of D-glucuronic acid and N-acetyl-D-glucosamine units linked through β-1,3 or β-1,4 glycosidic bonds and serves as a natural ligand for CD44 receptors expressed on macrophages. CD44 is a multifunctional receptor involved in intracellular, intercellular, and extracellular matrix interactions. The primary mode of HA binding to CD44 occurs via its NH2-terminal region located near the 135-amino acid domain of the receptor. Consequently, HA exhibits anti-inflammatory targeting by recognizing macrophage receptors. As a significant component of the vitreous humor, HA and its biocompatible derivatives are highly suitable for ocular delivery [29–31]. HA-CS nanocomplexes loaded with siRNA could penetrate the rabbit vitreous, and following intravitreal injection, a reduction in laser-induced neovascularization in the rabbit retina was observed, accompanied by good tolerability, biosafety, and enhanced bioavailability [32]. HA serves as an excellent drug carrier in the treatment of ocular diseases, exhibiting outstanding drug diffusion and delivery properties. These attributes enable its wide-ranging application potential in intraocular drug delivery.

#### Cyclodextrins

Cyclodextrins are macrocyclic structures characterized by a cone-shaped, hollow, cylindrical morphology. The hydrophilic exterior of cyclodextrins is formed by secondary and tertiary hydroxyl groups at the larger and smaller openings, respectively, while the interior cavity is hydrophobic due to shielding by C–H bonds. This hydrophobic cavity can accommodate various organic compounds, forming inclusion complexes and modifying the physical and chemical properties of the encapsulated substance. Wang et al. synthesized nanomaterials containing brinzolamide inclusion complexes and hydroxypropyl-β-cyclodextrin complexes, prolonging drug release and enhancing the efficacy of brinzolamide eye drops in glaucoma treatment [33]. Cyclodextrins offer significant advantages in the treatment of ocular diseases, including drug protection, enhanced solubility, reduced toxicity, improved drug stability and enhanced drug delivery. These properties establish cyclodextrins as crucial components in the treatment of ocular diseases, playing a vital role that cannot be overlooked.

#### Natural medicine-based polysaccharides

Natural medicine-based polysaccharides exhibit unique and highly effective biological functions for treating ocular afflictions. For instance, *Lycium barbarum* polysaccharides (LBPS) ameliorated dry eye syndrome, mitigated oxidative damage in human trabecular meshwork cells, and maintained retinal and ganglion cell functionality [34–38]. In human corneal fibroblasts (HCFs), these polysaccharides reduced the formation of pro-fibrotic proteins following in vitro corneal injury and suppressed the expression of IL-8 and IL-6, thereby acting as prophylactic medication before corneal refractive surgery [39]. Moreover, LBPS demonstrated anti-Aβ_1-40_ oligomerization properties, inhibited NLRP3 inflammasome activation, and exerted anti-apoptotic effects, alleviated inflammation and cellular pathology in vitro age-related macular degeneration (AMD) models [40]. *Astragalus* polysaccharides (APS) protected ARPE-19 cells, a spontaneously arising retinal pigment epithelium (RPE) cell line, and rat primary RPE cells under high glucose conditions through miR-182/Bcl-2 and miR-204/SIRT1 signaling pathways, restraining mitochondrial damage, endoplasmic reticulum (ER) stress and cell apoptosis [41, 42], ultimately improving diabetic retinopathy (DR) RPE cell function. *Ginkgo biloba* leaf-derived polysaccharides (PGBL) reduced tumor necrosis factor-α (TNF-α) expression in the aqueous humor of endotoxin-induced uveitis (EIU) model rats, demonstrating notable efficacy in treating ocular inflammation and glaucoma [43, 44]. *Dendrobium candidum* polysaccharides (DCPS) inhibited proliferation and induced apoptosis of human corneal epithelial cells (HCEC) under high glucose conditions, repairing HCEC damage [45]. Sodium alginate (SA), a natural polysaccharide extracted from brown algae such as kelp or sargassum, exhibits polyanionic behavior in aqueous solutions and possesses adhesive properties, serving as an adjunct in cataract surgery. Additionally, alginate oligosaccharides (AOS) treated D-galactose (D-gal)-induced SOD1, SOD2, and CAT protein expression in the lenses of C57BL/6J mice, decelerating lens damage and aging [46]. However, these polysaccharides display low absorption, poor bioavailability, and unstable chemical structures in ocular tissues. Therefore, combining polysaccharides and nanotechnology in disease treatment compensates for the inherent shortcomings of natural polysaccharides. For instance, when encapsulated within high molecular weight CS (HCS)-based nanogels, resveratrol exhibited no inflammatory or cytotoxic effects on ARPE-19 cells. After cellular internalization, researchers observed an endo-lysosomal escape of nanogels [47]. LBPS and DCPS were integrated with SA to fabricate nanomaterials, thereby enhancing drug compatibility and stability within the body [48]. These findings serve as a positive reference for the application of natural plant polysaccharides in conjunction with nanomaterials for the treatment of ocular disorders.

In conclusion, carbohydrate-based nanomaterials demonstrate significant potential in the field of ocular drug delivery, owing to their biocompatibility, biodegradability, and unique recognition properties. The development of these nanomaterials has the potential to revolutionize the treatment of various ocular diseases, such as dry eye syndrome, glaucoma, DR, and AMD, by enhancing drug bioavailability, prolonging drug release, and improving therapeutic efficacy. Further research and development of carbohydrate-based nanomaterials are essential to unlocking their full potential and translating their benefits into clinical applications.

### Lipid-associated nanomaterials

Lipid-associated nanomaterials, a class of nanomaterials comprising lipids, are highly sought after for applications in diverse fields such as medicine, biotechnology, and materials science. This category includes liposomes, nanoemulsions, and lipid NPs, all of which possess unique properties that make them well-suited for targeted drug delivery, imaging, and diagnostic purposes. These lipid-based nanomaterials can be tailored to exhibit specific properties, such as size, shape, and surface charge, rendering them highly versatile and effective in various applications. Additionally, they can self-assemble and form complex structures, a characteristic that is particularly attractive for drug delivery and tissue engineering applications. Lipid-associated nanomaterials also demonstrate excellent biocompatibility, biodegradability, and low toxicity, making them ideal candidates for biomedical applications.

Liposomes are spherical vesicles composed of phospholipid bilayers capable of encapsulating both hydrophilic and hydrophobic drugs. These nanoscale structures are highly versatile and can be engineered to possess specific properties, such as size and surface charge, to enhance their drug delivery capabilities [49]. Liposomes exhibit biocompatibility, biodegradability, and non-toxicity, making them suitable for biomedical applications [50]. They can protect encapsulated drugs from degradation and clearance by the immune system, leading to improved drug efficacy and reduced side effects. Furthermore, liposomes can selectively target specific tissues or cells, improving drug delivery and reducing off-target effects. They have been employed in various applications, including cancer therapy [51], vaccine delivery [52], gene therapy [53], and cosmetic formulations [54]. Researchers continue to explore new formulations and modifications of liposomes to enhance their effectiveness and applicability across diverse fields.

Nanoemulsions are a type of nanomaterial consisting of small droplets of one liquid dispersed within another liquid. The droplets in nanoemulsions typically have a diameter ranging from 20 to 200 nm [55], making them highly stable and suitable for various applications, including drug delivery, food science, and cosmetics. Nanoemulsions can be engineered to possess specific properties, such as size, surface charge, and stability, rendering them highly versatile and effective in different applications. They can also be designed to exhibit specific drug release kinetics, crucial for achieving optimal therapeutic effects. One significant advantage of nanoemulsions is their ability to enhance the solubility and bioavailability of poorly water-soluble drugs. Encapsulating these drugs in nanoemulsions can improve their absorption and distribution within the body, leading to increased drug efficacy. Nanoemulsions are also highly stable and can be formulated to resist aggregation and coalescence, which can reduce their effectiveness. They can be functionalized with targeting ligands, such as antibodies or peptides, to selectively target specific tissues or cells for improved drug delivery. Due to their transparency, uniform texture, and comfortable application, nanoemulsions are gaining clinical significance in ophthalmology. Kang et al.’s prospective double-blind study revealed that a novel 0.05% Cyclosporin A topical nanoemulsion demonstrated superior lipophilicity and water solubility, effectively improving conjunctival inflammation and ocular symptoms in dry eye patients compared to a conventional emulsion [56]. This study provides a basis for the effective utilization of nanoemulsions in ocular drug delivery, demonstrating their potential in the field of ophthalmology.

**Fig. 1:**
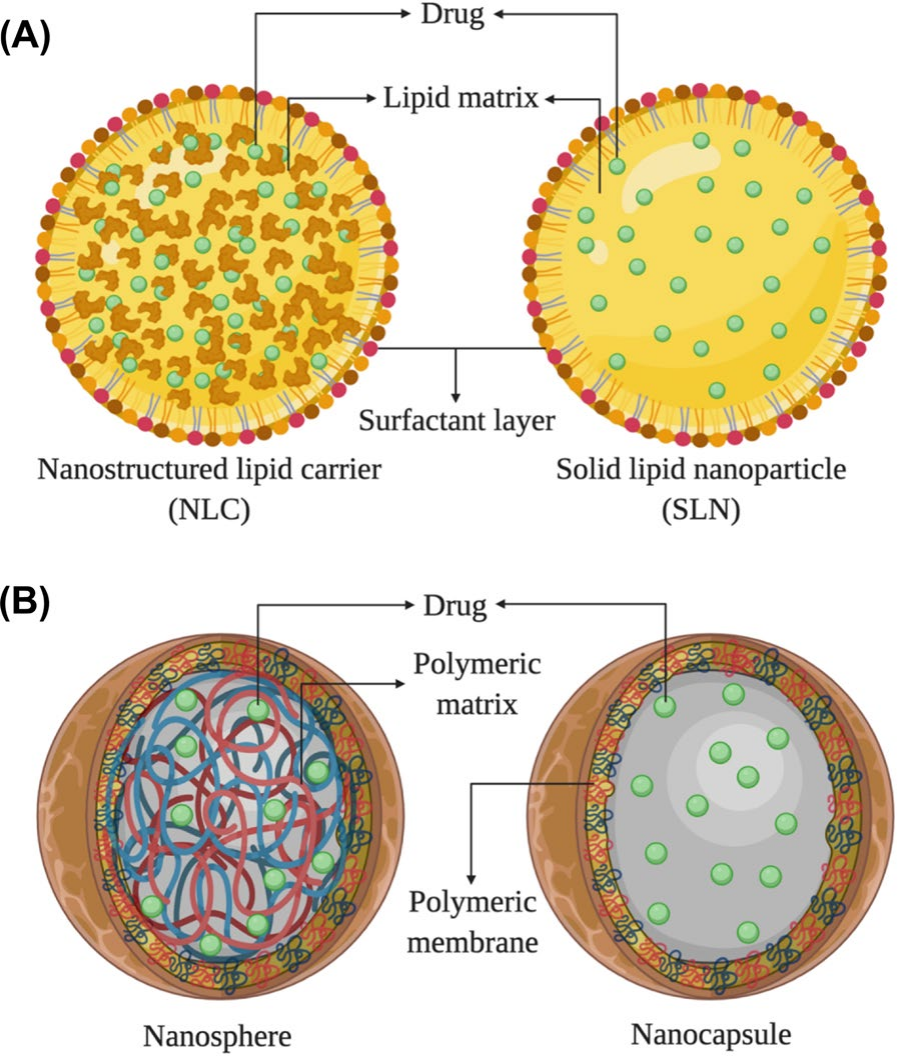
**A** Schematic overview of the structural organization of first-generation SLNs and second-generation lipid NPs-NLCs. **B** The structural design of nanocapsules and nanospheres. This is reprinted from Ref. [342] with permission from MDPI

Lipid NPs consist of lipophilic matrices and aqueous phases with particle sizes ranging from 100 to 1000 nm. Lipid NPs can be categorized into two developmental stages: first-generation solid lipid nanoparticles (SLNs) and second-generation nanostructured lipid carriers (NLCs) [57], both exhibiting similarities in biocompatibility and biodegradability. Lipid NPs have the unique property of being able to encapsulate both hydrophilic (water-soluble) and hydrophobic (lipid-soluble) substances [58]. This versatility is due to their composition, which includes lipids that possess both hydrophilic and hydrophobic regions. These lipids can form self-assembled structures, such as liposomes or lipid NPs, which can accommodate and entrap a wide range of drug molecules, regardless of their solubility properties [59]. The ability to encapsulate both hydrophilic and hydrophobic substances make lipid NPs suitable for a broad spectrum of drugs, enabling their effective delivery. This characteristic is advantageous in large-scale production because a single lipid-based nanoparticle formulation can accommodate different types of drugs, simplifying the manufacturing process and reducing the need for multiple formulations [58, 60]. SLNs originate from O/W emulsions, substituting liquid lipids in emulsions with lipid matrices such as fatty acids and fatty alcohols, rendering them solid at room temperature. SLNs decrease surface tension between lipid and water interfaces. Clinically, most drugs display low solubility; thus, combining them with SLNs results in more soluble medications for enhanced absorption. Surfactant coatings preserve stability, offering higher physical stability for SLN-based nanocarriers compared to nanoemulsions when solid structures are enveloped in stable surfactant layers. Drug-SLN binding methods can be classified into three distinct models based on drug distribution within SLNs: The homogeneous matrix model, drug-enriched shell model, and drug-enriched core model [61]. The second-generation lipid NPs, NLCs, were designed to overcome the limitations of first-generation SLNs. In comparison to SLNs, NLCs demonstrate high drug loading capacity, reduced aqueous content in particle suspensions, and minimal potential drug leakage during storage [62] **(**Fig. 1A**)**. So far, literature reports have shown that both NLCs and SLNs exhibit the ability to encapsulate small-molecule substances, enabling easier delivery to various ocular tissues. However, further research is needed to investigate the delivery of large-molecule substances such as peptides and proteins to the ocular region, particularly in the case of SLNs. Lipid-drug conjugates (LDCs) represent a new class of compounds generated through the lipophilic modification of water-soluble or poorly soluble drugs. Although SLNs and NLCs are appropriate for lipophilic drugs, their encapsulation efficiency for water-soluble drugs is quite low. This can lead to inadequate ocular drug delivery permeation and the inability to administer high doses of proteinaceous and peptide-based drugs. LDCs tackle these challenges by modifying drugs to boost absorption and therapeutic efficacy. Typically, LDCs are formed through the covalent bonding of water-soluble drugs or compounds that are challenging to formulate with lipids, thereby enabling lipophilic modification. This process imparts pharmaceutical properties to the drugs, including increased drug loading capacity, enhanced membrane permeability [63] and active transport, and improved drug bioavailability. Moreover, controlled release and targeted delivery can be achieved [64], minimizing toxic side effects [65]. Lipids such as fatty acids, glycerides, and phospholipids are commonly employed for conjugation with drugs. LDCs present a promising strategy for enhancing drug delivery for a wide array of therapeutic agents, including those with low solubility or poor permeability.

### Polymer Nanomaterials

Polymer nanomaterials constitute a class of nanomaterials comprised of synthetic or natural polymers with sizes typically ranging from 1 to 100 nm. Various types of polymer nanomaterials include polymeric NPs, polymer micelles, dendrimers, polymer hydrogels, and polymer nanofibers. These materials possess unique properties that render them highly valuable for diverse applications, including drug delivery, tissue engineering, and nanoelectronics. Polymer nanomaterials can be engineered with specific properties, such as size, shape, and surface chemistry, which can be customized for their intended application. They can also be functionalized with targeting ligands, like antibodies or peptides, to selectively target specific tissues or cells for enhanced drug delivery. A significant advantage of polymer nanomaterials is their ability to encapsulate a broad spectrum of drug molecules, encompassing both hydrophobic and hydrophilic drugs. This can improve the solubility and bioavailability of these drugs, resulting in better therapeutic outcomes and fewer side effects. Polymer nanomaterials can also be designed to respond to particular stimuli, such as changes in pH, temperature, or light, which can be useful for controlled drug release applications. Moreover, they can be engineered to be biodegradable or biocompatible, rendering them suitable for biomedical applications.

Polymeric NPs are structures that can carry drugs and proteins by covalently linking or adsorbing them to a polymer framework or surface [66]. They can take the form of nanocapsules or nanospheres and consist of natural or synthetic polymers [67] **(**Fig. 1B**)**. Examples of natural polymers include CS, heparin, HA, and starch, while synthetic polymers encompass polylactic-co-glycolic acid (PLGA), polyglycolic acid (PGA), and polyethylene glycol (PEG). Polymeric NPs provide a matrix-type solid colloidal particle that can facilitate drug release and targeted delivery in vivo, reducing toxic side effects. Nanospheres, with diameters ranging from 10–1000 nm, consist entirely of polymer materials with drugs encapsulated or adsorbed within them. By adsorbing surface-active agents, like poloxamine, onto nanosphere surfaces, NPs can evade recognition and degradation by the reticuloendothelial system (RES) in vivo, promoting drug circulation within the body [68]. PEA microspheres containing DEX were injected into rabbit eyes to observe drug metabolism within the vitreous humor, and the results revealed a sustained drug effect lasting up to three months [69]. Nanocapsules, conversely, possess an oily liquid core and an enveloping polymer shell that can incorporate drugs into the oily core or adsorb them onto the polymer surface, rendering this approach suitable for hydrophobic drug delivery [70]. Astragaloside-IV loaded into lipid nanocapsules (ASIV-LNCs) could reach the retinal layer via topical eye drops to treat AMD, demonstrating the feasibility of delivering nanocapsule-encapsulated drugs to the retinal layer using eye drops [71]. In summary, polymeric NPs offer various advantages, such as increased drug solubility, innovative drug administration methods, enhanced active ingredient utilization, and reduced cytotoxicity.

Polymer micelles, with diameters typically ranging from 10–100 nm, emerged as one of the most effective drug carriers in the 1990s [72]. These micelles are primarily spherical, featuring hydrophilic heads and hydrophobic tails, offering an advantage in the incorporation and transport of numerous hydrophobic drugs. The loaded drugs can encompass hydrophobic small molecules and negatively charged macromolecular nucleic acids (DNA and siRNA). Interactions between hydrophobic small molecules facilitate their integration into the micelle interiors. When incorporating negatively charged nucleic acid macromolecules, longer nucleic acids provide more binding sites with micelles, leading to increased drug stability. Polymer micelles exhibit remarkably low cytotoxicity in vivo because, following the disintegration of drug-loaded micelles, individual polymer chains are formed that can be excreted through renal metabolism [73]. Presently, polymer micelles have become one of the most extensively utilized drug carriers in the treatment of ocular diseases, providing exceptional tissue permeability upon contact with ocular tissues. Most notably, these polymer micelles possess high water solubility, enabling the production of transparent eye drops that neither interfere with vision nor compromise user comfort [74].Polymer hydrogels represent a class of three-dimensional, highly hydrophilic polymeric networks formed by water-soluble or hydrophilic polymers through chemical and physical interactions. They can be categorized into synthetic hydrogels, polysaccharide-based hydrogels, and peptide (protein)-based hydrogels. Synthetic hydrogels comprise polymers such as alcohols, acrylic acids, and their derivatives, including polyacrylic acid. Polysaccharide hydrogels encompass starch, cellulose, alginate, HA, CS, and others, while peptide-based hydrogels consist of collagen and poly-L-lysine. Due to their exceptional biocompatibility, environmental sensitivity, abundant sources, and cost-effectiveness, natural polymer hydrogels are extensively employed in biomedicine. André et al. discovered that biopolymeric hydrogels based on high-molecular-weight alginate and HA could serve as human vitreous substitutes, exhibiting high optical transparency and viscosity similar to vitreous. In vitro experiments revealed no cytotoxic effects on human fibroblasts, ARPE-19, and photoreceptor cells [75].

Polymer dendrimers are a class of highly branched, monodisperse polymers characterized by tree-like structures, formed by the linear connection of low molecular weight polymers via branching units. Dendrimers typically comprise a core, main polymer chains, and side chains of branching units. They exhibit precise control of physicochemical properties, extensive internal cavity structures, and densely functionalized surfaces. By adjusting the structure of the branching units and the distance between the main polymer chains, diverse dendrimer configurations can be prepared, facilitating improved combinations with various drugs for delivery. Commonly synthesized dendrimer components include polyamidoamine (PAMAM), poly(L-lysine) (PLL), polyethylenimine (PEI), and poly (propylene imine) (PPI). In a mouse model of oxygen-induced retinopathy (OIR), Generation-4 hydroxyl polyamidoamine dendrimer NPs were employed to deliver the drug triamcinolone acetonide (TA). Following intravitreal injection, dendrimer-conjugated TA (D-TA) was observed to inhibit retinal microglial inflammation, mitigating OIR-induced neuroretinal and visual function impairment [76]. This study demonstrates the effective approach and solution for the ocular administration of corticosteroids by reducing the dosage of corticosteroids through their conjugation with dendritic polymers. By coupling TA with dendritic polymers, the complications associated with the ocular use of corticosteroids can be minimized, offering a promising strategy for the proper use of corticosteroids in ocular applications.

Polymer nanofibers are elongated, slender fibers with diameters ranging from tens to hundreds of nanometers [77]. These fibers are generated via electrospinning, a process that involves applying an electric field to a polymer solution or melt, resulting in the formation of a jet that is subsequently stretched and solidified into nanofibers [78]. Polymer nanofibers possess a high surface area-to-volume ratio, offering enhanced mechanical properties, and high porosity, enabling their use as drug delivery systems. These materials have demonstrated promising results in drug delivery applications, particularly in the treatment of ocular diseases [79]. Nanofiber-based drug delivery systems provide improved drug loading capacity, sustained drug release, and targeted drug delivery, augmenting therapeutic efficacy while minimizing the risk of toxic side effects. Likewise, polymer nanofibers have been investigated for retinal tissue engineering, with studies utilizing electrospun nanofibers of biodegradable polymers like polycaprolactone and polylactic acid to create 3D scaffolds for retinal cell growth and differentiation [80]. Moreover, polymer nanofibers have also been deployed as drug delivery systems in ophthalmology, with electrospun nanofibers employed to encapsulate and deliver drugs directly to target ocular tissues. This approach has exhibited promise in treating diseases such as glaucoma and AMD [81, 82].

### Inorganic nanomaterials

Inorganic nanomaterials encompass NPs composed of inorganic substances, including metals, metal oxides, and semiconductors. These materials exhibit distinctive physical and chemical properties, rendering them promising candidates for biomedical applications [83].

Magnetic NPs constitute a type of inorganic nanomaterial characterized by unique magnetic properties. They are typically comprised of magnetic metals or metal oxides, such as iron, cobalt, nickel, and magnetite, with diameters ranging from 1 to 100 nm. For medical applications, magnetic particles must possess essential attributes, including non-toxicity, biocompatibility, injectability, and high accumulation in targeted tissues or organs. Presently, magnetic NPs are employed for cell sorting, targeted drug delivery and therapy, contrast agents for magnetic resonance imaging, and heating mediums for cancer thermotherapy. In ophthalmology, commonly used magnetic nanomaterials include superparamagnetic iron oxide nanoparticles (SPIONs) and gold-based magnetic materials. Among these, SPIONs represent a distinct class of nanomaterials composed of magnetite (Fe_3_O_4_) or maghemite (γ-Fe_2_O_3_), exhibiting a solid spherical shape. By aggregating with surfactants such as PEG, polyvinyl alcohol (PVA), and CS, SPIONs can form more stable and biocompatible nanomaterials [84]. During the fabrication process, SPIONs can be synthesized through microemulsion, hydrothermal, high-temperature pyrolysis, and chemical co-precipitation methods [85]. Complete drug delivery systems can be developed by encapsulating drugs with SPIONs and modifying their surfaces with surfactant materials, enabling targeted and precise drug therapy [86]. For instance, mesenchymal stem cells (MSCs) treated with SPIONs and intravenously injected into malnourished rat models demonstrated increased levels of glial-derived neurotrophic factor, ciliary neurotrophic factor, hepatocyte growth factor, and IL-10 in the rat retina compared to untreated MSC groups [87]. Moreover, due to their unique magnetic properties, SPIONs can induce temperature increases in local environments when exposed to magnetic fields, resulting in tumor cell death. Clinically, this therapeutic approach is referred to as magnetic nanomaterial thermotherapy. Dextran-coated iron oxide nanoparticles (DCIONs), upon magnetic field activation and at specific concentrations, could promote Y79 cell death by activating TNF-α activity in Y97 cells through the caspase-3/7 pathway. In the absence of a magnetic field, however, DCIONs displayed no cytotoxic effects on Y79 cells [88].

**Fig. 2:**
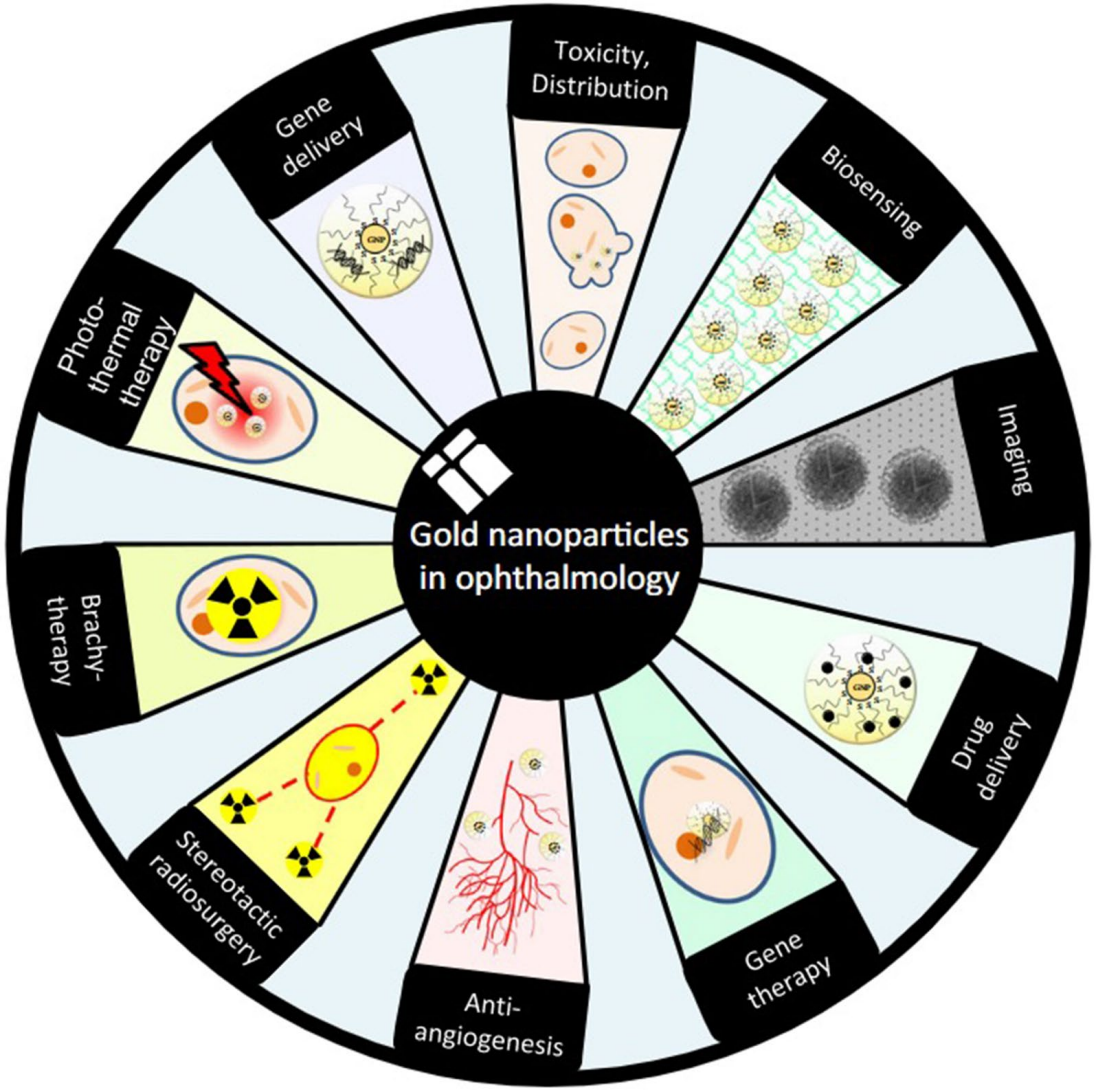
The diagram elucidates the multifaceted applications of AuNP in the domain of ophthalmology. This is reproduced from Ref. [23] with the authorization of John Wiley and Sons

AuNPs are a widely researched type of nanoparticle, with diameters typically ranging from 1 to 100 nm, exhibiting different colors based on their size. Owing to their stable physicochemical properties, large surface-to-volume ratio, and outstanding biocompatibility, AuNPs are well-suited for tumor targeting therapy, bioimaging, and as easily distinguishable identification markers in immunodetection and diagnosis due to their high density. In screening DR populations, color changes in AuNP-containing materials employed for urine testing can indicate diabetes progression. After photographing and analyzing these test strips with software systems, DR prevalence can be determined [89] **(**Fig. 2**)**.

## Inflammation in ophthalmology diseases

Inflammation is a prevalent factor in ophthalmic diseases, manifesting in a variety of symptoms, including redness, swelling, pain, and compromised vision. Inflammatory processes can impact various ocular structures, such as the cornea, iris, conjunctiva, choroid, retina, and optic nerve. This section aims to offer a comprehensive examination of inflammation in ophthalmic diseases, elucidating its etiology, clinical manifestations, and therapeutic approaches.

### Eyelid inflammation

Eyelid inflammation is primarily classified into four categories: hordeolum, blepharitis, viral palpebral dermatitis, and contact dermatitis [90]. Hordeolum is an acute, purulent or nodular inflammatory condition arising from eyelid glandular tissue infection by Staphylococcus aureus. Involvement of the meibomian gland results in a larger, deeper swelling within the eyelid, with the extent of swelling constrained by the tarsal plate. Conjunctival hyperemia and edema may be apparent. If the Zeis gland is affected, a smaller and more superficial swelling occurs near the eyelash base. Approximately four days post-hordeolum onset, the course of the condition depends on individual resistance, with Staphylococcus aureus reinfections within the lesion potentially spreading or remaining localized [91, 92]. Worsened inflammation may lead to eyelid cellulitis development, or an abscess may form, culminating in a firm, white nodule. Treatment strategies encompass cold and warm compresses, antibiotic eye drops, ultra-short-wave therapy, and more [93]. Severe cases or instances of eyelid cellulitis may necessitate oral or intramuscular antibiotics. Once the abscess is localized, incision and drainage can be performed for pus removal.

Blepharitis encompasses squamous, ulcerative, and angular forms [94]. Current research posits that chronic inflammation may be triggered by irritants resulting from the local degradation of sebum by Malassezia [95]. Treatment options include a 2% sodium bicarbonate solution for local cleansing, short-term antibiotic ointment use for mild symptoms, and systemic oral lipid antibiotics to reduce bacterial lipase production in severe cases. Ulcerative blepharitis, characterized by chronic or subacute purulent inflammation of eyelash follicles and associated glands [96], is typically caused by Staphylococcus aureus, epidermidis, or coagulase-negative Staphylococcus infections, primarily affecting immunocompromised children. Clinical treatment involves selecting appropriate medication following bacterial culture and drug sensitivity tests, with strategies including local warm compresses, secretion removal, and localized antibiotic application, with bacitracin as the preferred choice and long-term aminoglycoside use as an alternative [97]. Angular blepharitis originates from Moraxella, Staphylococcus aureus infections, or, rarely, vitamin B2 deficiency. Treatments include zinc sulfate eye drops (0.25–0.5% concentration) to inhibit Moraxella-produced enzymes, oral lipophilic antibiotics, and timely vitamin supplementation for individuals with vitamin B2 deficiency.

Viral palpebral dermatitis includes herpes simplex and herpes zoster forms, caused by herpes simplex virus type I and varicella-zoster virus infections, respectively. With weakened immunity, the virus can invade the eyelid, resulting in inflammation. Clinically, clusters of semi-transparent, yellowish pus-filled vesicles may emerge on the skin [98]. Pathological scraping tests can reveal multinucleated giant cells [99], while Giemsa staining may display acidophilic viral inclusion bodies, and peroxidase staining may yield positive results. Treatment options consist of topical zinc oxide and antibiotic ointments, local or systemic antiviral medications such as acyclovir, and intramuscular interferon injections, depending on the condition’s severity [100].

### Conjunctivitis

The conjunctiva, categorized into the bulbar, palpebral, and fornix conjunctiva based on location [101], encompasses a significant portion of the eye’s surface area. Its direct contact with the external environment renders it vulnerable to pathogenic factors, including bacteria, which may provoke inflammation and damage. Conjunctivitis may arise from microbial and non-microbial factors, as well as endogenous and exogenous factors. Infections can also disseminate from adjacent tissues, such as the nasal cavity. Microbial infections, encompassing bacterial, viral, chlamydial, fungal, and parasitic infections, constitute the most prevalent causes of conjunctivitis [102]. The condition is primarily classified into bacterial conjunctivitis, immune-mediated conjunctivitis, chlamydial conjunctivitis, and viral conjunctivitis. Fundamental clinical treatment approaches for conjunctivitis include antibiotic eye drops, ointment application, and systemic administration of antibiotics or sulfonamides [103].

### Keratitis

The cornea, an integral component of the eye’s refractive system, functions as the initial refractive medium for light entering the eye. Its convex, highly transparent structure is soft, avascular, and rich in sensitive nerve endings, rendering it essential for maintaining clear visual quality [104]. Keratitis, a primary cause of global blindness, is the primary reason behind corneal blindness in both developed and developing nations, with an approximate occurrence rate ranging from 2.5 to 799 cases per 100,000 population per year [105]. The disease’s etiology comprises microbial infections [106], spread from adjacent tissues, and autoimmune systemic diseases like rheumatoid arthritis. Based on causative factors, keratitis can be categorized into infectious, immune-mediated, malnutritional, and neurotrophic types. Infectious keratitis is most prevalent, marked by prominent symptoms such as photophobia, tearing, and ocular pain, along with varying degrees of vision loss. Primary treatments involve infection control, inflammation reduction, ulcer healing promotion, and scar formation minimization. Depending on the causative agent, distinct medications are employed for various types of infectious keratitis [107]: topical or systemic antibiotics such as cefotaxime and tobramycin for bacterial keratitis; antifungal medications like natamycin eye drops for fungal keratitis [108]; acyclovir or ganciclovir eye gels, potentially combined with corticosteroids for inflammation control in herpes simplex virus keratitis [109]; and cationic inhibitors such as chlorhexidine bigluconate coupled with antifungal medications for Acanthamoeba keratitis [110]. During treatment, artificial tears like sodium hyaluronate drops can serve as adjunctive therapy for eye moisturization. Based on the depth of corneal infiltration, diverse surgical approaches can be employed, such as amniotic membrane transplantation or conjunctival flap coverage, lamellar or penetrating keratoplasty [111]. The emergence of commercial artificial corneas, including AlphaCor, Miok, and Boston II keratoprosthesis [112], offers optimism for patients with corneal diseases lacking corneal graft sources.

Dry eye syndrome, a distinct form of keratitis, constitutes a multifactorial disease characterized by tear film abnormalities and ocular discomfort, fatigue, and other unfavorable symptoms [113]. The pathogenesis of dry eye is intricate, encompassing immune-inflammatory response, apoptosis, and neurogenic inflammation, among other factors, which interrelate and amplify each other, ultimately leading to or exacerbating dry eye [114]. Current research suggests that hyperosmotic tear film and immune-mediated inflammation of the lacrimal gland are vital factors in the persistent development of dry eye [114]. Various cytokines, such as IL-1β, IL-17, TNF-α, IL-6, and tumor growth factor-γ (TGF-γ), play a significant role in dry eye pathogenesis [115]. Medications remain the primary treatment modality, and the field continues to be a focal point of ophthalmologic research. Currently available topical medications include artificial tears, CsA, autologous serum, corticosteroids, and tetracycline derivatives [116]. While artificial tears can alleviate mild dry eye symptoms and temporarily stabilize the tear film, they cannot reverse the progression of dry eye inflammation or halt the disease process. Treatment priorities for dry eye encompass reducing ocular surface inflammation (OSI), stimulating the growth and recovery of ocular surface epithelial cells, and enhancing lacrimal gland function. Targeting moderate to severe dry eye with anti-inflammatory treatment for ocular surface immune-mediated inflammation represents a novel direction in dry eye therapy [117, 118]. The local application of immunomodulators can improve dry eye-related signs and significantly reduce the expression of ocular surface inflammatory markers [119].

### Scleritis

The sclera, representing the outermost layer of the eyeball, is a robust and elastic dense white tissue primarily composed of type I collagen, proteoglycans, and minimal amounts of elastin and fibrillin proteins [120]. It features sparse blood vessels and nerves. When collagen fibers experience chronic inflammation, they become infiltrated by inflammatory cells, resulting in diffuse or nodular lesions that can involve surrounding tissues [121], causing keratitis and uveitis. Approximately 30% of scleritis patients exhibit systemic autoimmune diseases [122], necessitating collaboration with internal medicine physicians for diagnosis and treatment. Scleral inflammation is classified into episcleritis, which is the inflammation of the thin vascular connective tissue on the scleral surface, and scleritis, an inflammation of the scleral matrix layer arising from collagen fiber destruction and cellular infiltration by inflammatory factors [123]. Inflammatory types predominantly involve type IV delayed or type III immune complex-mediated hypersensitivity reactions. Treatment options, contingent on severity, may encompass topical corticosteroid eye drops, oral NSAIDs, immunosuppressants, and periocular TA injections to alleviate inflammation. In instances of extensive lesions, autologous lamellar scleral grafting or allogeneic scleral transplantation may be required [124]. Although scleral transplantation can bring significant benefits in certain cases, there are also limitations and challenges to consider. These include a lack of donor sources, immune rejection reactions, surgical complications, postoperative recovery, and suboptimal outcomes. It is essential for physicians to assess the feasibility of transplantation and weigh the pros and cons based on the individual patient’s specific condition and needs in order to formulate the most suitable treatment plan.

### Uveitis

The uvea, a crucial component of the eyeball and one of the most vascularized tissues, is situated adjacent to the sclera and retina. It consists of the iris, ciliary body, and choroid, connecting the anterior and posterior segments of the eye. Due to its unique anatomical structure, inflammation is classified based on location: anterior uveitis, intermediate uveitis, posterior uveitis, and panuveitis [125, 126]. Inflammation typically propagates from the front to the middle, while posterior inflammation generally spreads forward, encompassing the entire uveal tissue. In rare instances, it may extend to adjacent tissues, causing inflammatory glaucoma, vitritis, and retinitis [127].

Uveitis is categorized into infectious and non-infectious types based on the cause. Infectious uveitis further divides into endogenous and exogenous types [128]. Exogenous uveitis results from direct invasion by bacteria, fungi, and viruses, while endogenous uveitis arises from antigen–antibody and complement system responses to pathogens. Autoimmune factors involve antigens such as melanocyte-associated antigens and retinal S-antigens, instigating pathological changes through T helper cell 17 (Th17)-derived inflammatory cytokines like IL-23 and IL-17 [129]. Trauma-related factors activate arachidonic acid, generating prostaglandins and thromboxane A2 via cyclooxygenase and leukotrienes through lipoxygenase, leading to uveitis [130]. Immune genetic factors have linked various types of uveitis to HLA antigens, with HLA-B27-positive ankylosing spondylitis patients being susceptible to uveitis [131] and Vogt-Koyanagi-Harada syndrome correlating with HLA-DR4 positivity [132]. Based on the findings of these studies regarding the association between HLA and ocular inflammatory diseases, testing for HLA genotypes in patients can aid in predicting the risk and type of uveitis. For individuals at high risk, regular eye examinations and early intervention are crucial for early detection and treatment of uveitis.

Treatment options encompass ciliary muscle paralytics such as M-receptor blockers like atropine and tropicamide for mydriasis and relief from ciliary and sphincter muscle spasms [133]. Corticosteroids, including DEX and prednisone, are the primary medications for uveitis in Western medicine. Topical corticosteroid eye drops can be employed for localized anterior uveitis, while systemic oral or intravenous administration is reserved for severe cases [134]. Antibiotics sensitive to the causative agent should be utilized for infectious uveitis. NSAIDs like diclofenac sodium and indomethacin, which inhibit prostaglandins and suppress inflammatory responses, can be employed [135]. Given that immune reactions contribute to uveitis pathogenesis, combined corticosteroid and immunosuppressive therapy (e.g., methotrexate) may be considered for recurrent cases [136, 137]. Intermediate and panuveitis with vascular lesions and macular edema can be treated with intravitreal corticosteroid injections (e.g., TA or Ozurdex) combined with laser or cryotherapy [138, 139], while surgical excision of the affected tissue may be required in severe cases.

### Retinitis

Inflammatory retinal disorders originate from infectious and non-infectious sources, as well as inflammation in the systemic or nearby tissues extending to the retina. Conditions in this category include cytomegalovirus retinitis (CMVR), retinal vasculitis, DR, and AMD [140]. A prime example of an infectious retinal inflammatory condition is CMVR, which is the predominant ocular opportunistic infection in AIDS patients and a leading cause of blindness [141]. During the initial phase of cytomegalovirus infection, viral DNA is introduced into the nuclei of uninfected retinal cells, instigating viral DNA transcription and the production of viral particles, thereby initiating an immune response [142, 143]. This triggers retinal inflammation, characterized by yellow-white necrotic lesions interspersed with red hemorrhages along blood vessels, radiating from the posterior pole to the periphery. Diagnosis involves detecting cytomegalovirus antigen PP65, CMV-mRNA, CMV isolation, or inclusion bodies [144]. Elevated intraocular IL-8 and mannose-binding lectin (MBL) levels also hold diagnostic significance in CMV infection. Ganciclovir, sensitive to CMV, is typically administered intravenously or through intravitreal injection [145], and vitrectomy is performed in the presence of complications such as preretinal membranes and proliferative vitreoretinopathy.

Retinal vasculitis, a vascular injury disease mediated by immune complexes, arises from autoimmune or infectious factors [146]. Frequently affecting both arterioles and venules, it presents as flame-shaped hemorrhages of varying sizes, dot-like and blotchy hemorrhages, tortuous blood vessels accompanied by white sheathing, and late-stage retinal neovascularization and vitreous hemorrhage. Fluorescein fundus angiography (FFA) serves as the gold standard for diagnosing retinal vasculitis [147]. Treatment depends on the specific condition: patients with mild retinal vasculitis without macular cystoid edema, significant vitreous inflammation, or severe ischemic alterations on FFA may not require treatment but need close monitoring. Macular cystoid edema can be treated with intravitreal anti-vascular endothelial growth factor (VEGF) medications [148] or DEX implants [149]. Retinal ischemia and non-perfusion capillaries necessitate retinal laser photocoagulation to eliminate ischemic regions. Infectious retinal vasculitis calls for the identification of the responsible microorganism and targeted anti-infective therapy. Surgical intervention is warranted when retinal detachment or significant vitreous hemorrhage occurs that is incapable of independent absorption, provided inflammation is managed with medication.

DR, a retinal disorder triggered by chronic hyperglycemia, exhibits a strong association with inflammation in its progression. Key indicators include increased retinal vascular permeability, infiltration of inflammatory cells, and expression of inflammatory and chemotactic factors, ultimately leading to retinal tissue deterioration, capillary degeneration, and neovascularization [150]. Takeuchi et al. observed significantly elevated expression levels of inflammatory cytokines IL-4, IL-6, IL-17A, IL-21, IL-22, and TNF-α in the vitreous cavity of patients with proliferative diabetic retinopathy (PDR) compared to the patients’ own serum concentrations and higher than the concentrations in the vitreous cavity of patients with epiretinal membranes or macular holes [151]. Clinically, macular edema resulting from DR can be treated with Ozurdex administered into the vitreous cavity [152].

AMD is a prevalent retinal degenerative disease affecting central vision and a leading cause of blindness in individuals over 50. Pathological features primarily manifest as the loss of RPE and the degeneration of photoreceptor cells. The intricate pathogenesis involves inflammation, hypoxia, oxidative stress, edema, and the disease’s development is accompanied by neovascularization and macular edema [153]. Liu et al. [154] detected significantly elevated expression levels of IL-17 in the serum of 23 AMD patients compared to age-matched healthy individuals. Biopsy of local retinal tissue in AMD patients also revealed increased expression levels of retinal IL-1β and IL-23. These studies indicate the involvement of inflammatory cytokines in the pathogenesis of AMD. Corticosteroids play a unique role in the treatment of AMD by inhibiting the pro-angiogenic effects of inflammatory cytokines and targeting extracellular components of choroidal neovascularization [155], such as inflammatory cells and fibroblasts. Due to the complexity of AMD pathogenesis, combined treatment (corticosteroids + anti-VEGF drugs) is a logical approach to address the disease progression mechanism. Vakalis et al. observed a reduction in retinal thickness following intravitreal injections of DEX combined with bevacizumab [156]. Kiernan et al. posited that combined therapy was superior to standard anti-VEGF treatment in cases of exudative AMD unresponsive to standard treatment, reducing the number of intravitreal injections and stabilizing or improving visual acuity [157]. The combined treatment approaches proposed in these studies undoubtedly yield better results for AMD compared to monotherapy. However, there is no single method that can perfectly cure AMD without adverse reactions. Natural products may be safer than synthetic chemicals and have simpler administration routes, as they have been used for the treatment of diseases for a long time, with many being suitable for oral administration. However, more research and effort are needed to determine their ability to penetrate the blood-retinal barrier (BRB) and their metabolic rates within the eye.

### Optical neuritis

Optic neuritis (ON) comprises a group of inflammatory diseases affecting the optic nerve and represents one of the prevalent neuro-ophthalmic disorders encountered in clinical practice [158]. ON is primarily classified into multiple sclerosis-related optic neuritis (MS-ON), neuromyelitis optica-related optic neuritis (NMO-ON), and infection-related ON.

MS-ON is an inflammatory demyelinating disease of the nervous system, with a majority of ON patients concurrently experiencing MS [159]. The two conditions are closely intertwined, with ON signifying the ocular manifestation of MS. The principal pathogenic mechanisms involve the loss of myelin sheaths and a relative reduction in nerve cells. Activation of autoreactive T cells, B cells, and macrophages releases cytokines, causing inflammation [160]. Infiltration of inflammatory cells into neuronal cells leads to oligodendrocyte death-mediated demyelination, activation of neuroglial cells (including microglia and astrocytes), and axonal degeneration [161, 162]. Pathological changes in ON lesions resemble those in chronic inactive MS plaques [163, 164], with each neural lesion exhibiting characteristics of long-term damage. NMO-ON, also known as Devic’s disease, preferentially affects the optic nerves and spinal cord, involving unilateral ON, brainstem, cerebral, and diencephalic syndromes [165]. The pathogenesis is associated with antibodies against astrocyte water channel protein 4 (AQP4-IgG) or MOG [166]. Current research concentrates on mitigating astrocyte damage and necrosis, as well as oligodendrocyte damage and demyelination. AQP4-IgG binds to astrocyte foot processes, activating complement, antibody-dependent cell-mediated cytotoxicity, and complement-induced eosinophil degranulation, resulting in severe central nervous system inflammation and astrocyte damage. Furthermore, AQP4-IgG binding to AQP4 receptors disrupts astrocyte transcellular water transport or receptor internalization [167]. By regularly monitoring the levels of AQP4-IgG, the progression of the disease can be assessed, enabling clinicians to adjust treatment plans and take appropriate intervention measures to control the inflammatory response. This provides patients with more accurate prognostic assessment and management strategies. Infection-related ON, induced by various pathogenic microorganisms, elicits immune-mediated ON, serving as a precipitating factor for MS-ON. Other optic neuropathies are associated with autoimmune disorders such as systemic lupus erythematosus (SLE), Sjögren’s syndrome, autoimmune thyroiditis, and myasthenia gravis [168, 169], often coinciding with NMO-ON. Current treatments and research aim to suppress such inflammatory cascades and alleviate symptoms. Clinically, high-dose corticosteroid therapy (oral, intravenous, and periocular injections) significantly improves patients’ visual acuity, while immunosuppressants like methotrexate reduce the recurrence rate of ON [170]. Other treatment options include plasma exchange, intravenous immunoglobulin, antibiotics, and neurotrophic medications.

## Anti-inflammatory properties of nanomaterials in ophthalmology diseases

**Fig. 3:**
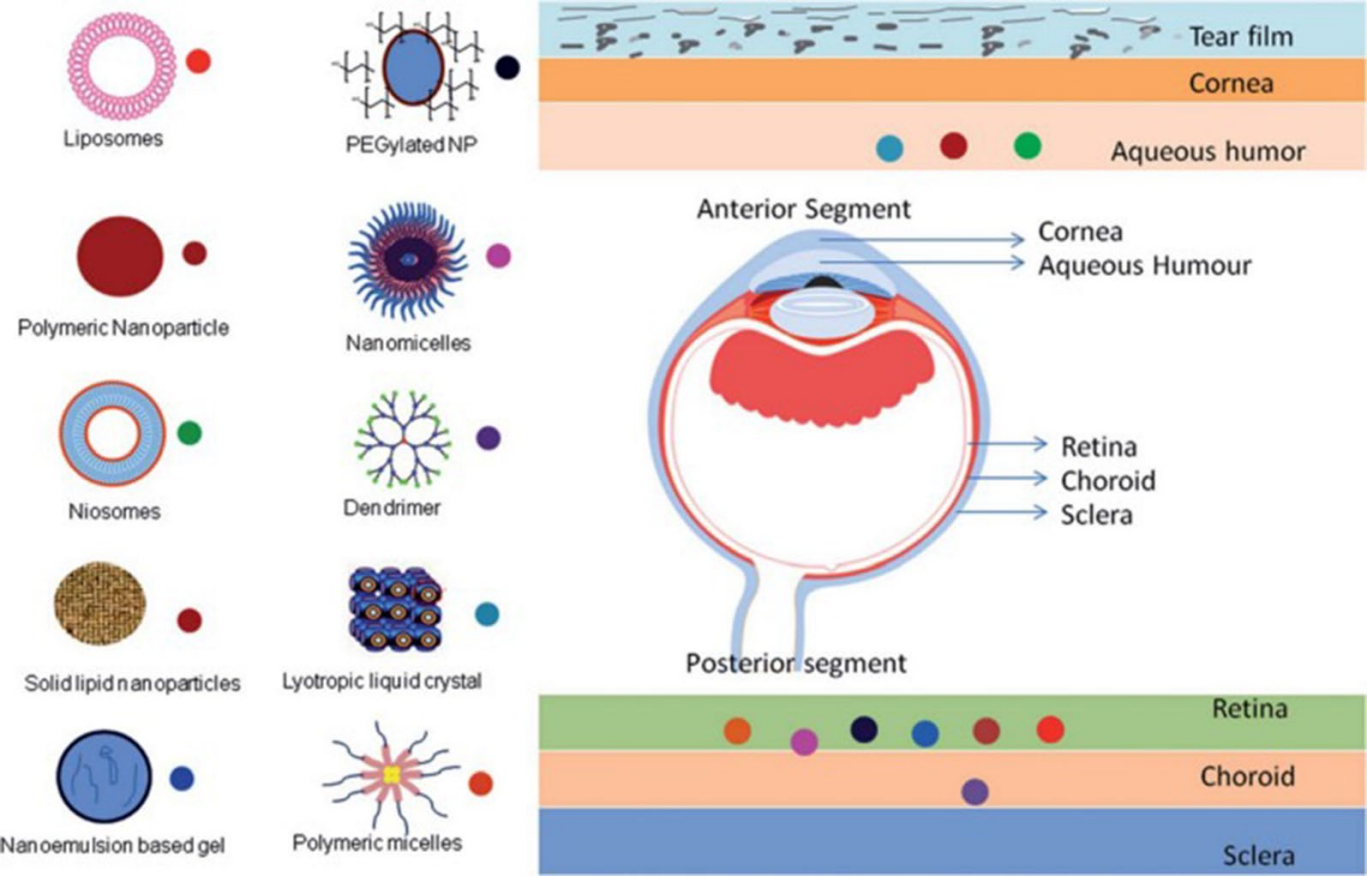
The image illustrates the varying capacities of different nano-formulations to traverse distinct barriers and reach diverse tissues within the eye, as dictated by their individual properties. This is referenced from Ref. [343], reproduced with permission from Royal Society of Chemistry Advances

Nanomaterials, characterized by their small size and high surface area, have gained considerable interest in various fields, including biomedicine. One area of particular interest is their potential anti-inflammatory properties, which can be attributed to their interactions with biological systems, such as cells, tissues, and whole organisms. In this discussion, we will explore common types of nanomaterials with anti-inflammatory properties and their potential applications in ophthalmic diseases **(**Fig. 3**)**. Recent research suggests that the anti-inflammatory properties of nanomaterials can be attributed to two distinct mechanisms: the nanoknife mechanism and the electron transfer mechanism. The nanoknife mechanism refers to the sharp-edged structure of nanomaterials, which can puncture the cell walls of microorganisms such as bacteria, causing cellular disruption, dysfunction, and ultimately leading to the death of microorganisms. The electron transfer mechanism involves charge transfer between nanomaterials and bacteria, resulting in the oxidation and damage of essential cellular structures or components. Positively charged NPs can alter the function of the electron transport chain within bacteria, extracting electrons directly and causing oxidative stress in lipoproteins and other substances on the bacterial cell wall, thereby inhibiting bacterial growth and producing anti-inflammatory effects. Literature reports that ZnO-NPs, Ag-NPs, graphene materials (GMs), nanoceria, and nano-flower structured MoS_2_ exhibit antibacterial properties through this mechanism [171–173].

Ocular bandages, encompassing natural amniotic membrane variants and synthetic alternatives, serve a vital role in treating ocular injuries [174, 175]. Electrospun fibrous membranes (EFMs) are employed as synthetic wound dressings due to their facile production and accessible sources [175]. Nonetheless, their use in ophthalmic applications is restricted, as they lack antibacterial capabilities [176, 177]. Recently, silver nanoparticles (Ag-NPs) have been extensively integrated into medical material scaffolds for their exceptional antibacterial properties. Yan and colleagues coated Ag-NPs onto EFMs and poly (lactic acid) (PLA) composite scaffolds, which significantly impeded the growth of Escherichia coli, Staphylococcus aureus, and Fusarium spp. in bacterial culture dish experiments [178]. Consequently, Ag-NPs coated EFMs and PLA composite scaffolds exhibited potential for treating fungal and bacterial keratitis by promoting corneal and conjunctival epithelial cell proliferation, inhibiting elevated expression of inflammatory factor IL-6, and facilitating wound healing. This study paves the way for the development of advanced biomaterial-based strategies for ocular tissue engineering, offering a promising solution for improving ocular cell proliferation and combating infections in the field of ophthalmology. Cai et al. discovered that inorganic cerium oxide NPs (nanoceria) exhibited antioxidative properties, rendering them suitable for endogenous reactive oxygen species (ROS) scavengers with enzyme-mimetic catalytic activity [179]. These enzymes encompass superoxide dismutase (SOD), hydrogen peroxide enzymes, peroxidases, and oxidases. Additionally, research has unveiled the anti-inflammatory effects of nanoceria, which, following intravitreal injection, not only downregulated VEGF expression and inhibited neovascularization [180] and the expression of inflammatory factors IL-3 and IL-7 [181] in VLDLR^*−/−*^ mice but also suppressed Müller cell gliosis in mouse retinal tissue via the JNK/NF-κB signaling pathway [182]. Qian et al. observed that nanoceria attenuated inflammatory corneal lesions in rat models and in vitro HCECs by inhibiting IKB/NF-κB-mediated inflammatory responses through the suppression of oxidative stress [171]. These findings indicate that nanoceria may constitute a novel therapeutic strategy for managing ocular inflammatory neovascular diseases. Carbon nanostructured materials, including carbon nanotubes (CNTs) and graphene, are distinctive nanomaterials boasting exceptional biocompatibility and mechanical stretchability. They have demonstrated the ability to maintain the elasticity and rigidity of collagen fibers for treating corneal lesions while exhibiting good biocompatibility in the eye without evidence of active inflammation upon blue Alcian staining [183]. Lin et al. devised a remote monitoring and treatment system for chronic OSI utilizing carbon nanostructured materials, comprising a smart contact lens and a thermotherapy eye patch [184]. Graphene, a carbon nanomaterial, possessed outstanding electrical conductivity, enabling a graphene field-effect transistor (FET) to remotely monitor the OSI biomarker MMP-9 concentration in tear fluid via a smartphone [185]. A diagnosis of OSI is established when the concentration surpasses 200 ng/ml. Transparent, stretchable eye patches were fabricated using Ag-NPs and an elastomer film (polydimethylsiloxane). These patches adhere to the eye during the application, utilizing the exceptional thermal conductivity and stable mechanical deformation resistance of Ag-NPs to deliver thermotherapy for OSI treatment. This approach has shown promising therapeutic results in both animal experiments and human trials [184].

## Nanomaterial-based drug delivery systems are a promising approach for the treatment of ophthalmology diseases

Nanomaterial-based drug delivery systems have emerged as a highly promising approach for the treatment of ophthalmology diseases due to their numerous advantages (Table 1). The unique properties of nanomaterials enable precise control over drug release, enhanced drug stability, improved bioavailability, and targeted delivery to specific ocular tissues. These systems utilize nanoparticles or nanocarriers to encapsulate drugs and protect them from degradation, ensuring their efficacy and prolonged shelf life. By employing nanotechnology, ophthalmology drugs can be administered using non-invasive routes such as eye drops, minimizing patient discomfort and improving treatment adherence. Furthermore, nanomaterials facilitate the penetration of drugs across ocular barriers, allowing them to reach the target tissues more effectively [186]. Additionally, the ability to design nanocarriers with surface modifications enables targeted delivery to specific areas of the eye, reducing systemic exposure and potential side effects [187–189]. The versatility of nanomaterial-based drug delivery systems also allows for combination therapy, wherein multiple drugs or therapeutic agents can be co-delivered, enabling comprehensive treatment of complex ophthalmology conditions. With ongoing advancements in nanotechnology, these innovative drug delivery systems hold immense potential to revolutionize the treatment of ophthalmology diseases, offering improved therapeutic outcomes and enhancing the quality of life for patients.

**Table 1:** Advantages of nanotechnology for ocular drug delivery

Advantages	Descriptions
Enhanced drug bioavailability	Nanoparticles used in ocular drug delivery can increase drug solubility, stability, and permeability, leading to improved bioavailability and therapeutic efficacy
Prolonged drug release	Nanocarriers can be designed to release drugs in a sustained and controlled manner, prolonging the therapeutic effect and reducing the need for frequent administration
Targeted delivery	Nanoparticles can be surface-functionalized with ligands that specifically bind to ocular tissues, allowing targeted drug delivery to the desired site, such as the cornea, conjunctiva, or retina. This minimizes systemic exposure and reduces side effects
Protection of drugs	Nanocarriers provide protection to drugs from degradation by enzymes and other physiological factors present in the ocular environment, ensuring drug stability and prolonged shelf life
Improved ocular penetration	Nanoparticles can enhance drug penetration across ocular barriers, such as the cornea and blood-retinal barrier, enabling drugs to reach the target tissues more effectively
Reduced frequency of administration	The prolonged release and enhanced bioavailability offered by nanotechnology allow for reduced dosing frequency, improving patient compliance and convenience
Minimized toxicity	Nanoparticles can encapsulate drugs, reducing their toxicity and enhancing their safety profile. Moreover, targeted delivery minimizes systemic exposure, further reducing the potential for systemic side effects
Non-invasive delivery	Nanotechnology-based ocular drug delivery systems offer non-invasive administration routes, such as eye drops or ophthalmic gels, avoiding the need for injections or invasive procedures
Combination therapy	Nanotechnology enables the co-delivery of multiple drugs or therapeutic agents within a single nanocarrier, allowing for combination therapy to address complex ocular diseases or conditions
Potential for personalized medicine	Nanoparticles can be customized with specific drug formulations, release profiles, and targeting ligands, enabling personalized treatment approaches tailored to individual patient needs

### The delivery of glucocorticoids

Glucocorticoids, or corticosteroids, comprise a class of steroid hormones naturally produced by the adrenal glands and can be artificially synthesized for medical applications. They are frequently employed in ophthalmology to address various inflammatory eye conditions, such as uveitis, scleritis, and ON, by suppressing the immune system and diminishing inflammation. Corticosteroids serve as potent inhibitors of the phospholipase A2 (PLA2) enzyme, which can curtail the synthesis of arachidonic acid. As a precursor of numerous inflammatory mediators, the judicious use of corticosteroids in ocular inflammatory diseases can effectively suppress the inflammatory response and avert diverse complications.

Multiple studies have investigated the employment of nanomaterial-based drug delivery systems for targeting ocular tissues and achieving sustained corticosteroid release **(**Table 2**)**. For instance, Alami et al. utilized polycaprolactone-polyethylene glycol-polycaprolactone (PCL-PEG-PCL) micelles loaded with DEX to treat endotoxin-induced anterior uveitis in rabbits, demonstrating that DEX-loaded micelles could mitigate inflammation and attain the maximum therapeutic effect within 36 h [190]. Likewise, polymeric TA NPs prepared using PLGA polymer had proven effective in decreasing the expression of inflammatory factors NO and PGE2 in a rabbit anterior uveitis model induced by endotoxin [191]. Furthermore, polymer micelles and nanomicelles, prepared using various monomers and surfactants, have been explored for uveitis treatment. These delivery systems have been observed to maintain a prolonged effective drug concentration in the choroid and retina while significantly reducing ocular inflammation in rabbits. For example, Pradip et al. employed cationic NLCs of the drug triamcinolone acetonide (cTA-NLC) to treat anterior uveitis, demonstrating sustained drug release for up to 24 h [192]. Nanoemulsion eye drops have also received approval from the US FDA for ocular disease treatment. Difluprednate (DFAB or Durezol), a nanoemulsion eye drop developed by Sirion Therapeutics, has found widespread use in treating anterior scleritis due to its ability to penetrate the scleral barrier and access the uveal tissue [193]. Mahmoud et al. discovered that Durezol exhibited more potent anti-inflammatory activity than prednisolone in controlling inflammation, reducing corneal edema, clearing anterior chamber cells (ACs), and maintaining stable intraocular pressure in patients undergoing cataract surgery [194]. In summary, nanomaterial-based drug delivery systems hold significant promise for treating ocular inflammatory diseases using corticosteroids.

**Table 2:** The application of nanomaterials in combination with corticosteroids in the treatment of ocular diseases

Glucocorticoids	Nanomaterials	Size (nm)	Production method	Cells (in vitro)	Animals (in vivo)	Administration route	Characteristics and effects	Refs.
DEX	PCL-PEG-PCL nanomicelles	40.04 ± 2.42	Film hydration method	L929, STF and isolated corneas of bovines	LSP-induced anterior uveitis in rabbits	Eye drops	DEX-loaded PCL-PEG-PCL nanomicelles exhibited robust biocompatibility and cellular tolerance, alleviation of the clinical symptoms of uveitis with a delayed onset, comparable to commercially available suspension formulations	[190]
DEX	CH-MEs	< 200	Water titration method	STF and cellulose membrane system	Endotoxin-induced uveitis in rabbits	Eye drops	DEX-loaded CH-Ms exhibited excellent mucoadhesive properties and stability, sustained drug release, improved anti-inflammatory activity, comparable to commercially available suspension formulations	[195]
DEX	MBA and TG-NIPAAM-VP-MAA polymeric nanomicelles	300–450	Free radical polymerization method	–	LSP-induced anterior uveitis in rabbits	Eye drops	DEX-loaded MBA and TG-NIPAAM-VP-MAA polymeric nanomicelles exhibited remarkable mitigation of uveitis symptoms and reduced inflammatory response within 48 h; DEX-loaded MBA-NIPAAM-VP-MAA polymeric micelles, characterized by their small particle size, exhibited strong adhesion, favorable curative effect, and potent and prolonged anti-inflammatory activity	[196]
DEX	P40S and P80 nanomicelles	14.5 ± 0.4	Rotary evaporation method	STF and cellulose membrane system	Rabbits	Eye drops	DEX-loaded P40S and P80 nanomicelles exhibited strong stability, non-irritating, achieved effective drug concentration in retinal and choroidal tissues	[197]
DEX	HA CS-NPs	400.57 ± 15.23	Ionotropic gelation method	STF	–	–	DEX-loaded HA CS-NPs exhibited a robust drug loading capacity and efficient drug release, along with excellent physical stability and enhanced adsorption to mucous membrane	[28]
DEX	γ-CD and β-CD microparticles	20.4 ± 10.3	–	–	Rabbits	Eye drops	DEX-loaded γ-CD and β-CD microparticles exhibited attainment of high concentrations in the vitreous and retina; DEX-loaded γ-CD microparticles exhibited excellent cellular tolerance and chemically stability	[198]
DEX	TA and egg LE nanomicelles	4.4 and 4.7	–	Human scleral cells	–	Iontophoresis	DEX-loaded TA and egg LE nanomicelles exhibited increased water solubility, sustained drug release, and enchaned delivery across the scleral barrier	[199]
DEX	SA-FFFE NPs	30	Classic solid-phase peptide synthesis method	RAW264.7, HCECs, and cellulose membrane system	Endotoxin-induced uveitis in rabbits	Eye drops	DEX-loaded SA-FFFE NPs exhibited sustained drug release, no observed cytotoxicity or eye irritation, and substantial inhibition of the secretion of NO, TNF-a, and IL-6	[200]
DEX	Pullulan NPs	326 ± 29	–	Mouse retinal organ culture and bovine vitreo-retinal organ culture	Rats, rabbits, and mice	Intravitreal injections	DEX-loaded pullulan NPs exhibited remarkable safety profiles, prolonged retention within the vitreous humor, and reduced frequency of intravitreal drug injections	[201]
TA	PLGA NPs	195	Modified emulsification and solvent diffusion method	–	LSP-induced uveitis in rabbits	Subconjunctival injection	TA-loaded PLGA NPs exhibited superior efficacy in reducing the inflammatory factors such as flare, cell, and fibrin, infiltrating cells, proteins, NO, and PGE2, compared to the microparticles of TA and PA	[191]
TA	Capmul^®^ MCM C10, soya LE, and Captex ^©^ 200 P NLCs	< 200	Hot microemulsion method	LPS-induced HCFs and isolated corneas of goats and pigs	–	Eye drops	TA-loaded NLCs exhibited robust corneal permeability, gradual drug release, remarkable safety, and reduced TNF-α levels	[192]
DFAB	Nanoemulsions	–	–	–	–	Eye drops	DFAB-loaded nanoemulsions exhibited alleviation of the clinical manifestations of scleritis, penetration of the scleral barrier to reach the uvea, reduced corneal edema, elimination of ACs, inhibition of inflammation, and maintaince of the stability of intraocular pressure	[193, 194]
FA	PAMAM dendrimers	3–10	Covalently conjugating fluocinolone acetonide to the dendrimer method	–	*Homozygous* *recessive* rdy albino RCS rats (prone to retinal degeneration)	Intravitreal injections	FA-loaded PAMAM dendrimers exhibited targeted inhibition of retinal microglia activation, increased viability of the photoreceptor outer nuclear cells, sustained drug release, and inhibition of retinal inflammation	[202]
Hydrocortisone	Albumin NPs and P80 nanomicelles	100 and 300	–	Isolated corneas of pigs	Proxymetacain HCL-induced inflammation in the precorneal area in rabbits	Eye drops	Hydrocortisone-loaded albumin NPs and P80 nanomicelles exhibited robust corneal permeability and prolonged retention within the inflamed conjunctival capsule	[203]
DFBA	Caster oil and P80 lipid emulsion	104.4	High pressure emulsification method	–	Rabbits	Eye drops	DFAB-loaded lipid emulsion exhibited physical stability, high permeability within intraocular environment, and elevated drug concentrations in the aqueous humor	[204]
Netilmicin sulphate, DEX alcohol and phosphate	PHEA-PEG, PHEA-PEG-C_16_, and PHEA-C_16_ nanomicelles	10–30	–	BCEC and BcoEC	Rabbits	Eye drops	Drug-loaded PHEA-C_16_ and PHEA-PEG-C_16_ nanomicelles exhibited remarkable corneal permeability; Drug-loaded PHEA-PEG-C_16_ nanomicelles exhibited superior corneal permeability and enhanced drug bioavailability	[205]
LE	HPMC/MC/ALG-HP-β-CD or HP-CD polymer gels	–	Kneading, freeze drying, and co-precipitation method	Cellulose membrane system	Histamine solution-induced allergic conjunctivitis in rabbits	Eye drops	LE-loaded HPMC-HP-β-CD polymer gels exhibited excellent stability, ocular bioavailability, and potent anti-inflammatory efficacy	[206]

*DEX* dexamethasone; *PCL-PEG-PCL* polycaprolactone-polyethylene glycol-polycaprolactone dimethacrylate; *CH-Mes*, chitosan-coated cationic microemulsions; *MBA* or *TG, N*,N-methylene bis-acrylamide and triethyleneglycol dimethacrylate as cross as cross-linking agents; *NIPAAM* N-isopropylacrylamide; *VP* vinyl pyrrolidone; *MAA* methacrylate; *P40S* polyoxyl 40 stearate; *P80* polysorbate 80; *HA CS-NPs* hyaluronic-acid chitosan-sodium tripolyphosphate nanoparticle; *Gamma CD* gamma-cyclodextrin; *TA *sodium taurocholate; *NPs* nanoparticles; *LE* lecithin; *Dex-SA-FFFE*, dexamethasone-peptide conjugate; *FA* fluocinolone acetonide; *LE* loteprednol etabonate; *BCEC* bovine conjunctival epithelial cell; *BcoEC* bovine corneal epithelial cells; *NLCs* nanostructured lipid carriers; *LPS* lipopolysaccharide; *HPMC* hydroxypropyl methylcellulose; *HP-β-CD* hydroxypropyl-β-cyclodextrin; *cTA-NLC* cationic nanostructured lipid carriers of the drug triamcinolone acetonide; *MC* methylcellulose; *ALG* alginate; *STF* simulated tear fluid

### The delivery of antibiotics and antiviral agents

Antibiotics and antiviral agents constitute a category of medications employed to combat bacterial and viral infections by either eliminating or inhibiting the growth of bacteria and viruses. Healthcare professionals frequently prescribe these medications, which can be administered orally, topically, or intravenously, depending on the type and severity of the infection. Currently, antibiotics are widely utilized in ophthalmology to address bacterial eye infections, including eyelid inflammation, conjunctivitis, corneal ulcers, and endophthalmitis. Topical antibiotics in the form of eye drops or ointments are often recommended for mild to moderate infections, while severe infections may necessitate intravenous antibiotics. Commonly used antibiotics in ophthalmology encompass fluoroquinolones, aminoglycosides, macrolides, and tetracyclines. Nanomaterial-based drug delivery systems have demonstrated potential in ophthalmology for treating microbial infections. Antibiotics or antiviral agents can be encapsulated within nanocarriers such as liposomes, dendrimers, and NPs to augment drug delivery and enhance treatment outcomes. These nanocarriers can safeguard the antibiotic from degradation and boost its penetration through ocular barriers, resulting in sustained release and improved efficacy. Research has indicated that antibiotic- and antiviral agent-loaded NPs can effectively treat ocular infections like bacterial keratitis and endophthalmitis while minimizing systemic side effects **(**Table 3**)**.

**Table 3:** The application of nanomaterials in combination with antibacterial and antiviral drugs in the treatment of ocular diseases

Antibacteria & antiviral drugs	Nanomaterials	Size (nm)	Production method	Cells (in vitro)	Animals (in vivo)	Administration route	Characteristics and effects	Refs.
Flucytosine	AuNPs and F6 nanoliposomes	135.1 ± 12.0	Thin film hydration method	Cellulose membrane system	*C. albicans*-induced fungal endophthalmitis in rabbits	Eye drops	Flucytosine-loaded AuNPs and recipe F6 nanoliposomes exhibited the highest intraocular penetration depth of intraocular penetration and a potent antifungal effect	[207]
VRC	PBA-CS-VE nanomicelles	113 ± 5	Ethanol injectionmethod	HCE-T cells and cellulose membrane system	*C. albicans*-induced fungal keratitis in rabbits	Eye drops	VRC-loaded PBA-CS-VE nanomicelles exhibited robust mucoadhesive properties, remarkable corneal penetration ability, and prolonged retention in the anterior ocular segment	[208]
VRC	Glyceryl behenate/capric caprylic triglyceride, P80, sorbitan trioleate, and cetylpyridinium chloride NLCs	250.2 ± 03.1	Microemulsion method	Isolated corneas of pigs and HET-CAM test	–	Eye drops	VRC-loaded NLCs exhibited a high drug encapsulation efficiency and enhanced drug delivery to the cornea	[209]
Fluconazole	Liposomes	–	Reverse-phase evaporation method	–	*C. albicans*-induced fungal keratitis in rabbits	Eye drops	Fluconazole-loaded liposomes exhibited a superior antibacterial effect compared to the conventional fluconazole solution	[210]
T-HCL	Isopropyl myristate/Miglyol 812, Tween 80/Cremophor EL, and polyethylene glycol 400 nanoemulsions gels	< 30	Water titration method	-	*C. albicans*-induced fungal keratitis in rabbits	Eye drops	T-HCL-loaded sterilized F31 formula in situ NE gel exhibited minimal eye irritation, elevated C_max_, extended time to reach T_max,_ prolonged MRT, and enhanced bioavailability	[211]
Ciprofloxacin	SA, DP, Soybean PC, CH, and Carbopol 940 liposomal hydrogel	–	Reverse-phase evaporation method	Isolated corneas of rabbits	–	Eye drops	Ciprofloxacin-loaded liposomal hydrogel composed of PC/CH at a molar ratio of 5:3 exhibited the highest encapsulation efficiency. Ciprofloxacin-loaded liposomal hydrogel composed of PC, CH, and SA at molar ratio 5:3:1 exhibited the best penetration effect in the cornea	[212]
Levofloxacin	Stearic acid, Tween 80, and sodium deoxycholate SLNs	2.237	Box-Behnken design optimization method	*E. coli* and *S. aureus*, solate corneas of goats, and HET-CAM test	–	Eye drops	Levofloxacin-loaded SLNs exhibited rapid attainment of effective drug concentrations in excised goat corneas, sustained drug release, no eye irritation, and inhibition of *Staphylococcus aureus* and *Escherichia coli*	[213]
OFX	COL, PEG 400, and glycerin NLCs	153.5 ± 2.3	High shear homogenization method	Isolated corneas of rabbits	Staphylococcus aureus-induced keratitis in rabbits	Eye drops	OFX-loaded NLCs supplemented with glycerol (Ins3_OFX_) exhibited enhanced biocompatibility, prolonged retention time in the eye, and increased C_max_	[214]
Daptomycin	CS-NPs	200	Ionotropric gelation method	STF	–	–	Daptomycin-loaded CS-NPs exhibited interactions with mucin to increase residence time in the eye and potent bacterial inhibition in in vitro buffer solution experiments	[215]
BSF	CTAB-CNLCs	98.04–230.12	Simple melt emulsification method	Conjunctival fibroblasts	–	Eye drops	BSF-loaded CTAB-CNLCs exhibited favorable penetration through the 3D tissue model, non-cytotoxicity, and physical stability	[216]
Moxifloxacin hydrochloride	HA-LCS-NPs	141.1 ± 4.29	Iionotropic gelation method	Isolated corneas of rabbits and cellulose membrane system	Rabbits	Eye drops	Moxifloxacin hydrochloride-loaded HA-LCS-NPs exhibited elevated bioavailability, minimal eye irritation, and high MRT, AUC_0-6 h_, and P_app_ values than those of commercially available products	[217]
Acyclovir	Cyclodextrin and PVP nanofibers	370–505	Water titration method	Artificial saliva	–	–	Acyclovir-loaded cyclodextrin nanofibers exhibited highly water solubility and rapid dissolution	[218]
Acyclovir and ciprofloxacin	PVP and PCL electrospun nanofibers	267–932	–	PK-Eye model	–	Intravitreal injections	Acyclovir and ciprofloxacin-loaded PCL electrospun nanofibers exhibited sustained drug release	[219]

*AuNPs* gold nanoparticles; *VRC* voriconazole; *F6* (phosphatidylcholine, cholesterol, Span 60, and stearylamine at a molar ratio of 1:1:1:0.15); *PBA-CS-VE* phenylboronic acid conjugated chitosan oligosaccharide-vitamin E copolymer; *CS* chitosan; *OFX* ofloxacin; *T-HCl* terbinafine hydrochloride; *BSF* Besifloxacin hydrochloride; *PC* phosphatidylcholine; *CH* cholesterol; *SA* stearylamine; *HA-LCS-NPs* hyaluronic-acid-modified lipid-polymer hybrid nanoparticles; *HA* hyaluronic; *MRT* mean residence time; *AUC* area under the curve; *P*_*app*_ apparent permeability coefficients; *COL* chitosan oligosaccharide lactate; *CTAB* hexadecyltrimethylammonium bromide; *CNLC* cationic nanostructured lipid carriers; *PVP* hydrophilic poly(vinylpyrrolidone); *PCL* poly(ε-caprolactone); *F31, Miglyol*^*®*^* 812, Cremophor*^*®*^* EL* polyethylene glycol 400 (1:2) and water (5, 55 and 40%, w/w, respectively); *HET-CAM* Hen’s egg test-chorioallantoic membrane; *PK-Eye model* two-compartment model designed to mimic the intraocular aqueous outflow

**Fig. 4:**
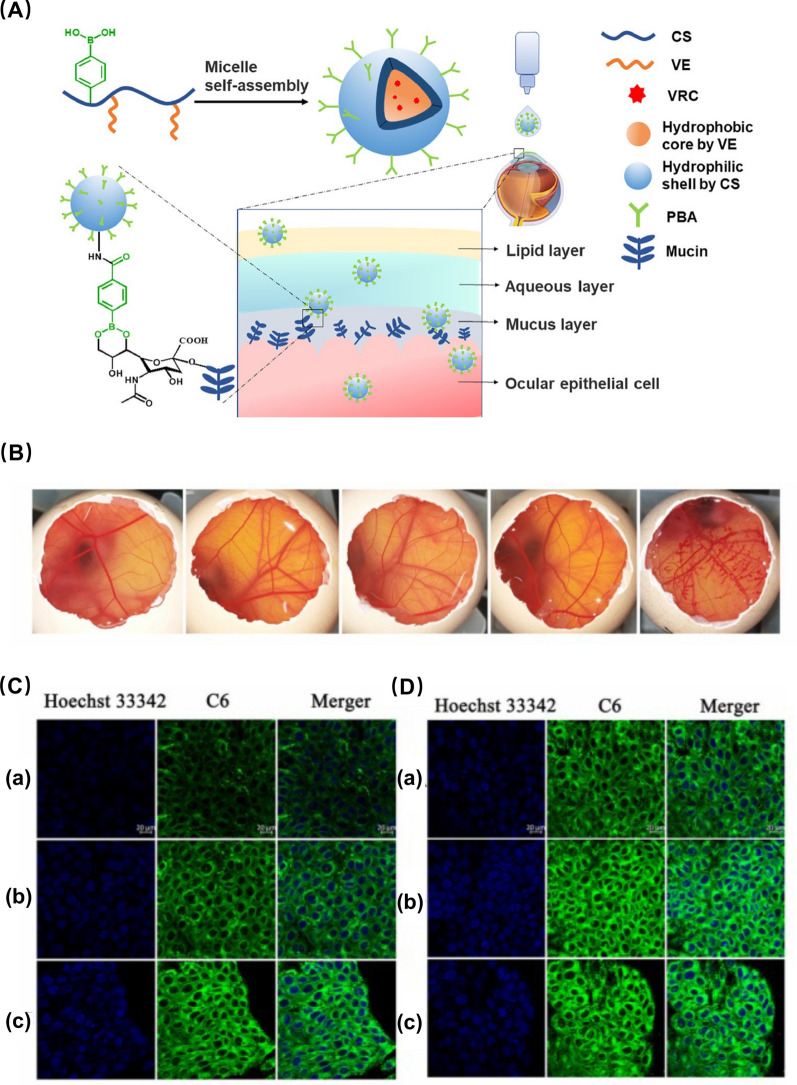
The effectiveness of PBA-CS-VE-VRC in treating fungal keratitis is illustrated, highlighting its role in minimizing ocular irritation while enhancing corneal permeability and extending immediate retention time for the administration of topical ocular medications. **A** The structure of PBA-CS-VE-VRC nanocelles and their role in treating corneal diseases is diagrammed. **B** The HET-CAM assay, an in vitro surrogate for ocular stimulation, is employed to assess the irritation potential of various preparations, namely: Sanitary saline, Sol-VRC, CS-VE-VRC, PBA-CS-VE-VRC, and 0.1 M NaOH solution on the chick embryo chorioallantoic membrane. **C** Fluorescent preparations of Sol-C6 (a), CS-VE-C6 (b), or PBA-CS-VE-C6 (c) were prepared and their respective uptake rates by the HCE-T cell line (human immortalized corneal epithelial cells) were observed using confocal fluorescence microscopy at 2 h and 4 h **D** intervals. Scale bar equals 20 μm. This figure has been reprinted from Ref. [208] with permission from Elsevier

**Fig. 5:**
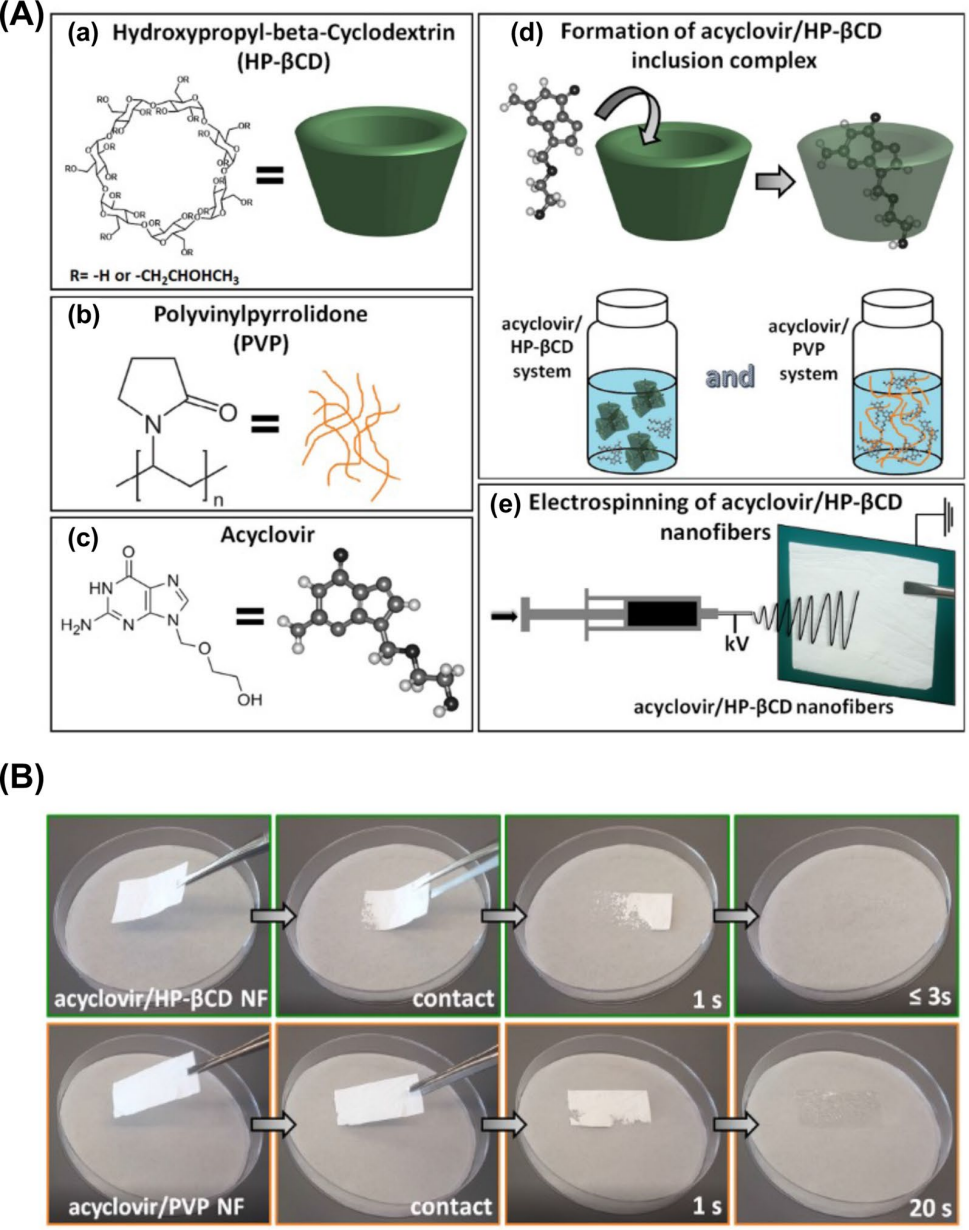
The electrospinning process for the creation of polymer-free and free-standing acyclovir/cyclodextrin nanofibers, notable for their exceptional histocompatibility and facilitation of drug release in the treatment of viral keratitis. **A** Chemical structures of (a) HP-βCD, (b) PVP, and (c) acyclovir are presented, (d-e) alongside schematic diagrams demonstrating their interrelationships. **B** Experimental data confirms the solubility of acyclovir/HP-βCD nanofibers and acyclovir/PVP nanofibers in an artificial saliva environment. Adapted from Ref. [218] with permission from Elsevier

Voriconazole (VRC) is a broad-spectrum antifungal agent utilized in ophthalmology for treating fungal keratitis [220] caused by Aspergillus or Candida species and endophthalmitis [221]. CS-VE-copolymer micelles modified with PBA form nanomicelles capable of specifically binding to sialic acid residues in mucin, extending the corneal residence time of the drug and enhancing VRC’s bioavailability and therapeutic efficacy for fungal keratitis [208] **(**Fig. 4**)**. Furthermore, Andrade et al. developed ocular administration of VRC based on cationic NLCs, which demonstrated effective drug concentration in corneal tissues within 30 min of application in vitro, playing a role in treating fungal keratitis [209]. Fluconazole, another broad-spectrum antifungal, was formulated into liposomes using the reverse-phase evaporation technique, yielding longer drug action time and quicker therapeutic effects in rabbit models of Candida keratitis compared to conventional fluconazole [210]. Nanofiber scaffolds provide not only structural and nutritional support but also deliver drugs or cells for eye implantation, facilitating drug dissolution and absorption. Acyclovir, an effective antiviral drug used for treating viral keratitis, has limited solubility and bioavailability; combining it with nanomaterials broadens its applicability. The development and application of electrospinning polymer-free, free-standing acyclovir/cyclodextrin nanofibers enhanced the solubility of acyclovir [218] **(**Fig. 5**)**. Hydrophilic PVP and slow-dissolving PCL form a fibrous membrane structure encapsulating acyclovir and ciprofloxacin, extending drug release in the eye [219]. This scaffold could function as an ocular implant, gradually releasing medication for treating vitritis and retinitis. Fluoroquinolones, a class of synthetic antimicrobial drugs including moxifloxacin, ofloxacin (OFX), ciprofloxacin, and levofloxacin, demonstrated exceptional activity against common Gram-positive and Gram-negative ocular pathogens [222, 223]. Combining these drugs with nanomaterials enhances their bioavailability in the eye. Hosny et al. prepared a liposomal hydrogel formulation containing ciprofloxacin. The mucoadhesive properties of the hydrogel matrix ensured close contact between liposomes and corneal epithelial cells, promoting drug penetration and preventing rapid elimination through tear circulation. The permeability of the liposome hydrogel was five times higher than that of the aqueous solution, and encapsulating ciprofloxacin prolongs its release. Such formulations decreased the dosing frequency in ocular inflammation treatment [212]. Levofloxacin-loaded SLNs optimized using Box-Behnken experimental design exhibited favorable therapeutic effects in treating conjunctivitis. Salman et al. found that NPs encapsulating the drug achieved a drug release rate of 0.2493 μg/cm^2^/h on excised goat corneas, extending drug release time and demonstrating excellent antimicrobial activity against Staphylococcus aureus and Escherichia coli-induced conjunctivitis [213]. OFX-loaded NLCs prepared by the high shear homogenization method, with glycerin as a plasticizer, exhibited increased bioadhesion, six times longer residence time in the anterior eye segment, and improved corneal inflammation and swelling in rabbits infected with Staphylococcus aureus within seven days compared to traditional formulations [214]. Daptomycin, a lipopeptide antibiotic, is employed to treat bacterial infections caused by Gram-positive bacteria such as Staphylococcus aureus and Streptococcus viridans, and it also exhibits some efficacy against drug-resistant strains. Silva et al. prepared CS NPs encapsulating daptomycin, proposing them as an ocular delivery system for the antibiotic to treat bacterial endophthalmitis, thereby enhancing local therapeutic effects and avoiding systemic drug toxicity [215].

In conclusion, nanomaterial-based drug delivery systems hold significant potential for improving the treatment of ocular inflammatory diseases by enhancing the solubility, bioavailability, and therapeutic efficacy of antibiotics. These advanced systems may lead to more effective and targeted treatments for a variety of ocular conditions.

### The delivery of nonsteroidal anti-inflammatory drugs

Nonsteroidal anti-inflammatory drugs (NSAIDs) represent a class of medications frequently employed to alleviate pain, diminish inflammation, and reduce fever. Their mechanism of action involves the inhibition of COX enzymes, which are instrumental in the synthesis of prostaglandins—chemical messengers implicated in the inflammatory response. In the field of ophthalmology, NSAIDs are utilized to address various conditions, including uveitis, OIS, and macular edema. They exert their effects by suppressing prostaglandin production, which in turn mitigates pain, redness, and swelling in the eye. Topical NSAIDs are favored in ophthalmology due to their rapid onset and localized therapeutic impact. Widely used NSAIDs in this domain encompass indomethacin, ketorolac, bromfenac, nepafenac, and diclofenac. Researchers have also investigated nanomaterial-based drug delivery systems for NSAIDs as a potential approach to treating ophthalmological diseases **(**Table 4**)**. These systems endeavor to address challenges linked to the topical delivery of NSAIDs to the eye, such as inadequate drug penetration, limited bioavailability, and brief residence time.

**Table 4:** The application of nanomaterials in combination with non-steroidal anti-inflammatory drugs (NSAIDs) in the treatment of ocular diseases

NSAIDs	Nanomaterials	Size (nm)	Production method	Cells (in vitro)	Animals (in vivo)	Administration route	Characteristics and effects	Refs.
Indomethacin	SLN, CS-SLNs and NLCs	226–265	Hot homogenization method	Valia-Chien cells and isolated corneas from Pel-Freez Biologicals	Rabbits	Eye drops	Indomethacin-loaded CS-SLNs exhibited enhanced ocular permeability and high drug concentrations in all tested eye tissues	[224]
Indomethacin	CS-NPs and CS-nanoemulsions	280 and 220–690	Spontaneous emulsification method		NaOH-induced corneal ulcers in rabbits	Eye drops	Indomethacin-loaded CS-nanoemulsions exhibited high drug concentrations within intraocular structures and moderate but significant inhibition of PMNL	[225]
Indomethacin	Poloxamer 188, Tween 80, and compritol^®^ 888 ATO SLNs	140	Hot homogenization method	Isolated corneas from Pel-Freez Biologicals	–	Eye drops	Indomethacin-loaded SLNs exhibited excellent chemical stability and increased corneal permeability	[226]
Ibuprofen	Pluronic^®^ F-127 NLCs	< 200	Melt- emulsification and ultrasonication method	Y-79	–	Eye drops	Ibuprofen-loaded NLCs with Pluronic® F-127 exhibited no observed cytotoxicity and sustained drug release	[227]
FB	Poloxamer 188 and Poly(lactic/glycolic) acid NPs	232.8 and 277.6	Solvent displacement method	Cellulose membrane system	SA-induced keratoconjunctivitis in rabbits	Eye drops	FB-loaded Poly(lactic/glycolic) acid NPs exhibited excellent embedding efficiency, favorable cellular tolerance in ocular tissue, anti-inflammatory efficacy, and sustained drug release	[228]
FB	COS-NLCs	77.9 ± 2.4	Melt-ultrasonic method	Isolated corneas of rabbits	Rabbits	Eye drops	FB-loaded COS-NLCs exhibited robust ocular bioadhesion, efficient corneal penetration, and slow drug clearance	[229]
Bromfenac sodium	ChS-CS-NPs	245.6 ± 14.22	Ionic gelation method	Isolated corneas of goats, STF and HET-CAM test	–	Eye drops	Bromfenac sodium-loaded ChS-CS-NPs exhibited remarkable corneal penetration, commendable corneal retention, and excellent intraocular tolerance	[230]
Dexibuprofen	PEGylated PLGA nanospheres	< 200	Solvent displacement method	Y-79, isolated corneas and scleras of rabbits, and cellulose membrane system	SA-induced ocular inflammation in rabbits	Eye drops	Dexibuprofen-loaded PEGylated PLGA nanospheres exhibited enhanced drug retention and penetration in the isolated corneal tissue, favorable cellular tolerance, and potent anti-inflammatory effects	[231]
Dexibuprofen	Poly (ethylene oxide)-poly (propylene oxide) polymeric nanomicelles	169.45	Direct equilibrium method	Cellulose membrane system	Rabbits	Eye drops	Dexibuprofen-loaded mixed polymeric nanomicelles exhibited enhanced drug solubility, no eye irritation, and favorable permeability	[232]
Diclofenac sodium	Homolipid and phospholipid SLNs	100–250	–	HENC and CEPI 17 CL 4	–	Eye drops	Diclofenac sodium-loaded homolipid and phospholipid SLNs exhibited high encapsulation efficiency, sustained drug release, and robust corneal permeability	[233]
Diclofenac	Polyvinyl alcohol liposomes	287	Calcium acetate gradient method	Cellulose membrane system	Rabbits	Eye drops	Diclofenac-loaded polyvinyl alcohol liposomes exhibited higher drug concentrations in the retina and choroid	[234]
Nepafenac	NP-NLC-Gel NPs	92.42 ± 3.46	Melt emulsification method	Isolated corneas of rabbits	Rabbits	Eye drops	Nepafenac-loaded NP-NLC-Gel NPs exhibited a high apparent permeability coefficient and a high drug concentration in tear fluid and aqueous humor	[235]
Diclofenac acid	Oil phase, Tween 80, and glycerin microemulsions	220–480	–	Cellulose membrane system	–	Eye drops	Diclofenac acid-loaded microemulsions exhibited excellent stability and sustained drug release	[236]
Ibuprofen	Gelucire 44/14 NLCs	80.6–160.1	Melted-ultrasonic method	Franz-type cells and isolated corneas of rabbits	–	Eye drops	Ibuprofen-loaded NLCs exhibited enhanced corneal permeability, high P_app_ and AUC values, and prolonged retention time in the cornea.	[237]

*SLNs* solid lipid nanoparticles; *FB* flurbiprofen; *SA* sodium arachidonate; *NSAIDs* nonsteroidal anti-inflammatory drugs; *FD-C* franz diffusion cell; *PMNLs* polymorphonuclear leukocyte infiltration; *NP-NLC-Gel* hydrogel modified nanostructured lipid carrier*; COS* chitosan oli gosaccharides; *ChS-CS-NPs* chondroitin sulfate-chitosan-nanoparticles

Indomethacin is a viable treatment for ocular inflammatory conditions such as conjunctivitis, uveitis, and other anterior segment inflammations. However, its poor solubility and stability present challenges in formulating topical ophthalmic solutions, as less than 5–10% of administered indomethacin reaches intraocular tissues. Prachetan et al. employed nanocarriers to encapsulate indomethacin and enhance its ocular penetration into posterior eye tissues [224]. The researchers developed indomethacin-loaded SLNs and NLCs and modified SLNs with CS chloride, a cationic water-soluble penetration enhancer. They assessed the in vitro release and in vivo distribution of the three formulations in corneal and sclera-choroid-RPE tissues. Results showed that indomethacin-loaded NLCs exhibited superior drug-loading capacity and elevated indomethacin levels within ocular tissues. Moreover, indomethacin-CS-SLN demonstrated enhanced permeation properties compared to indomethacin SLN [238, 239].

NLCs combined with the thermoresponsive polymer, Pluronic^®^ F-127, were formulated as eye drops to deliver ibuprofen with anti-inflammatory effects in the eye. This nanocarrier formulation exhibited excellent stability in Y-79 human retinoblastoma cells and extended the drug release profile of ibuprofen in the eye [227]. García et al. developed two different concentrations of dexibuprofen-loaded PEGylated PLGA nanospheres (0.5 and 1.0 mg/ml, with zeta potentials of -14.1 and -15.9 mV, respectively). Ex vivo experiments measured drug concentrations in the vitreous, aqueous humor, cornea, and sclera, revealing release curves lasting up to 12 h in both cornea and sclera. Notably, higher drug retention and permeability were observed in the ex vivo cornea. Cell viability assays, Hen’s egg test-chorioallantoic membrane (HET-CAM) test, and Draize tests confirmed the low cytotoxicity, non-irritating nature, and anti-inflammatory properties of dexibuprofen-loaded PEGylated PLGA nanospheres [231]. Vega et al. prepared poly(lactic/glycolic) acid NPs loaded with flurbiprofen (FB) and evaluated their anti-inflammatory effects in a rabbit ocular inflammation model induced by sodium arachidonate (SA) [228]. FB-loaded NPs effectively suppressed inflammation when administered 30 min before SA-induced inflammation and exhibited a longer residence time in inflamed eye tissues compared to healthy eyes. These findings suggest that FB-loaded PLGA-NPs possess excellent anti-inflammatory efficacy, potentially due to increased adhesion between the drug and biological cell membranes, prolonged drug residence time on the ocular surface, and sustained drug release from NPs.

Fujisawa et al. utilized the calcium acetate gradient method to encapsulate diclofenac within liposomes, achieving a 97% encapsulation efficiency [234]. In animal studies, this eye drop formulation resulted in a 1.8-fold increase in retinal-choroidal drug concentration compared to conventional diclofenac eye drops. Attama et al. used human corneal endothelial cells (HENC), stromal fibroblasts, and epithelial cells CEPI 17 CL 4 for bio-engineering human cornea construct (HCC) experiments, observing the permeability of diclofenac sodium-loaded SLNs on HCC. Analysis of permeation flux and permeation coefficients indicated superior corneal permeability per unit time and area, suggesting potential application for the prevention and treatment of preoperative and postoperative inflammatory responses in cataract surgery [233].

Bromfenac sodium eye drops are commonly used to treat conjunctivitis caused by various factors and to prevent inflammation preoperatively and postoperatively in ophthalmic surgeries. However, low corneal permeability, rapid tear turnover, and swift nasolacrimal drainage result in a short ocular residence time for bromfenac sodium. Tara et al. developed chondroitin sulfate-CS NPs encapsulating bromfenac sodium [230]. The formulation displayed a biphasic release curve, and compared to conventional eye drops, the permeation and corneal retention rates of bromfenac sodium were 1.62 and 1.92 times higher, respectively. The HET-CAM test was employed to assess the safety and drug toxicity of this formulation, demonstrating its non-toxicity and suitability for ocular drug delivery, with scores consistent with those of the saline group (negative control).

Lornoxicam (LX) is a selective COX-2 inhibitor used to treat various ocular inflammations and to alleviate postoperative inflammation and macular edema following cataract surgery [240]. However, LX is a hydrophobic drug, and its absorption and efficacy in the eye present significant challenges. Salama utilized a mixed micellar system made of poly (ethylene oxide)-poly (propylene oxide) to encapsulate LX [232], which was dissolved and encapsulated in the hydrophobic core of the micelles through hydrophobic interactions [241]. This approach aimed to improve permeation and increase residence time on the ocular surface to overcome ocular barriers [242]. Results indicated that the polymer micelles increased the solubility of LX from 0.0318 mg/ml to over 2.34 mg/ml, an enhancement of approximately 73-fold. In animal studies using rabbit eyes, histopathological examination and confocal laser scanning microscopy revealed the non-irritating nature and excellent penetration of the developed nanocarrier formulation on rabbit corneas. Consequently, polymer micelles encapsulating LX prolonged the drug’s residence time on the ocular surface and improved corneal permeability.

In conclusion, nanomaterial-based drug delivery systems hold promise for enhancing the delivery and efficacy of NSAIDs in treating various ophthalmic diseases. Nonetheless, further research is required to optimize these systems and evaluate their safety and efficacy in clinical settings.

### The delivery of immunosuppressants

Immunosuppressants represent a category of pharmaceuticals designed to attenuate or diminish the potency of the body’s immune response. These medications are frequently prescribed to inhibit the immune system from attacking transplanted organs or tissues in transplant recipients, as well as to address autoimmune diseases, such as those affecting the eyes. For severe inflammatory eye conditions, particularly in instances of various uveitis where corticosteroid therapy proves ineffective or is contraindicated due to systemic illness or dependence, immunomodulatory drugs are utilized to alleviate the adverse consequences of prolonged corticosteroid use. Notable immunomodulators encompass antimetabolites, alkylating agents, T-lymphocyte inhibitors like CsA and rapamycin, and biological agents. Presently, numerous nanomaterial-based drug delivery systems have been established for the targeted and controlled release of immunosuppressants, encompassing liposomes, polymeric NPs, hydrogels, dendrimers, and micelles **(**Table 5**)**.

**Table 5:** The application of nanomaterials in combination with immunosuppressive drugs in the treatment of ocular diseases

Immunosuppressive drugs	Nanomaterials	Size (nm)	Production method	Cells (in vitro)	Animals (in vivo)	Administration route	Characteristics and effects	Refs.
CsA	PLA-b-Dex-NPs	26.9–29.1	Nanoprecipitation method	–	Rabbits and scopolamine-induced dry eye in mice	Eye drops	CsA-loaded PLA-b-Dex-NPs exhibited prolonged retention in the eye, potent anti-inflammatory effects, restoration of ocular surface goblet cells, and reduced dosage and frequency of medication compared to conventional preparation	[243]
CsA	Compritol 888 ATO, poloxamer 188, and Tween 80-SLNs	225.9 ± 5.5	High shear homogenization method	–	Rabbits	Eye drops	CsA-loaded SLNs exhibited high drug concentrations in the aqueous humor, without causing any ocular irritation	[244]
CsA	Cys-NLCs	66.9 ± 0.4	Melt-emulsification method	Cellulose membrane system	Rabbits	Eye drops	CsA-loaded Cys-NLCs exhibited sustained drug release, strong bioadhesion, and elevated AUC_0-24 h_ and MRT_0-24 h_ values in aqueous humor, tear fluid and ocular tissue	[245]
CsA	INS and MS	385	–	–	Rabbits	Eye drops	CsA-loaded INS exhibited minimal eye irritation; CsA-loaded MA exhibited no eye -irritation; CsA-loaded INS and MA exhibited a higher drug concentrations in the cornea compared to commercially available formulations	[246]
Rapamycin	Vit E TPGS and Oc-40 nanomicelles	10.84 ± 0.11	Novel solvent evaporation method	rPCECs and D407	Rabbits	eye drops	Rapamycin-loaded MNFs exhibited high drug encapsulation efficiency, favorable cell tolerance, elevated drug concentration in the retina-choroid, and no drug residue in the vitreous	[247]
TAC	NH_2_-PEG-b-PLA and HPMC nanomicelles	101.4 ± 1.3	Solvent-evaporation-induced self-assembly in aqueous solution method	HCECs, isolated corneas of rabbits, and cellulose membrane system	Rabbits and allogeneic penetrating corneal transplantation in rats	Eye drops	TAC-loaded nanomicelles exhibited a high permeability, sustained high concentrations, and prolonged anti-rejection effect in ocular tissues of rat corneal transplantation	[248]
TAC	mPEG-b-PLGA nanomicelles	81.3 ± 1.3	Solvent-evaporation-induced self-assembly in aqueous solution method	HCECs, isolated corneas of rabbits, and cellulose membrane system	Rabbits and Allogeneic penetrating corneal transplantation in rats	Eye drops	TAC-loaded mPEG-b-PLGA nanomicelles exhibited a higher corneal permeability than that of 0.05% tacrolimus eye drops, excellent biological safety, reduced expression of NFAT, CD4, and CD8 in tissues following corneal transplantation, and inhibition of immune rejection	[249]
TAC	PLGA-NPs	164– 375	Emulsification-diffusion method	Isolated corneas of rabbits	Rabbits	Eye drops	TAC-loaded PLGA-NPs exhibited remarkable corneal permeability, excellent stability, superior tissue tolerance, and high drug concentrations in the cornea, conjunctiva, and aqueous humor	[250]
TAC	Gellan gum NPs	274.46 ± 8.90	Improved ionotropic gelation method	Isolated corneas of goats, HET-CAM test, and cellulose membrane system	Rabbits	Eye drops	TAC-loaded gellan gum NPs exhibited high encapsulation efficiency and loading capacity, prolonged drug release, extended residence time in the cornea, and the improved dry eye symptoms	[251]
TAC	MSNAPTES silica NPs	103 ± 14.2	Improved ionotropic gelation method	ARPE- 19 and HET-CAM test	Rats	Intravitreal injections	TAC-loaded MSNAPTES silica NPs exhibited excellent biocompatibility with no observed cytotoxicity.	[252]
TAC	N-palmitoyl-N-monomethyl-N, N-dimethyl-N, N, and N-trimethyl-6-O-glycol CS-NPs	200	Thin-film hydration method	–	Rabbits	Eye drops	TAC-loaded NPs exhibited excellent physical stability, highly permeability, no eye irritation, and high drug concentrations	[253]
TAC	PLGA, castor oil, Tween ^®^ 80, Cremophor ^®^ EL, and Lipoid^®^E80 nanocapsules	106–166	Solvent displacement method	Isolated corneas of pigs	S-antigen and pertussis toxin-induced EAU in rats, LPS-induced keratitis in mice	eye drops	TAC-loaded nanocapsules exhibited excellent physical stability, high permeability, no eye irritation, inhibition of KC, MIP-2, IL-6 and GCSF expression	[254]
TAC	Compritol^®^ 888 ATO, GMS, Tween-80, and glycerin SLNs in situ gel	122.3 ± 4.3	Probe sonication method	Cellulose membrane system	Rabbits and ovalbumin-induced immune conjunctivitis in mice	Eye drops	TAC-loaded SLNs in situ gel exhibited sustained drug release, inhibition of inflammatory mediators from conjunctival mast cells, and suppression of OVA-specific IgE, IFN-γ and IL-4	[255]

*CsA* cyclosporine A; *PLA-b-Dex* poly(D,L-lactic acid) and *dextran; TAC* tacrolimus; MSNAPTES 3-aminopropyltriethoxysilane; *rPCECs* rabbit corneal epithelial cells; *D407* human retinal pigment epithelial cells; *Vit E TPGS* vitamin E tocopherol polyethylene glycol succinate; *MS* micellar solution; *Oc-40* octoxynol-40; *mPEG-b-PLGA* methoxy poly (ethylene glycol)-block-poly (D, L)-lactic-co-glycolic acid; *NFAT* nuclear factor of activated T cells; *CD4* differentiation cluster 4; *CD8* differentiation cluster 8; *INS* an in-situ nanosuspension; *MS* a micellar solution; *MNFs* rapamycin-loaded mixed nanomicellar formulations

Research has demonstrated that 0.05% CsA can effectively address dry eye syndrome [256–258]. To develop NP drug carriers with enhanced hydrophilicity, density, and stability, scientists have formulated poly (D, L-lactic acid) and dextran (PLA-b-Dex) NPs [243]. These NPs had been surface-modified with PBA to adhere to carbohydrates on ocular mucosa via covalent bonding with cis-diol groups [259]. The efficacy of CsA-loaded PBA-modified NP formulations was compared to Restasis^®^ in induced mouse and rabbit dry eye experiments. The investigation revealed that the NPs delivered a near-infrared fluorescent dye to the eye for over 24 h, while the free dye was predominantly cleared from the ocular surface within 3 h. Following one month, NP eye drop formulations containing 0.005–0.01% CsA reduced corneal lymphocytes and polymorphonuclear leukocytes, mitigated inflammation symptoms, and aided in the recovery of ocular surface goblet cells. In contrast, administering Restasis to mice three times daily did not restore ocular surface goblet cells. The mucoadhesive nanoparticle eye drop platform extended ocular surface retention time and effectively treated dry eye while reducing the total CsA dosage by 50 to 100 times, thus diminishing side effects and lengthening the dosing interval. CsA encapsulated in lipid-based nanomaterials such as SLN and NLC has been extensively investigated for ocular treatments. Researchers loaded CsA into SLNs and administered it to rabbit eyes in vivo [244]. Aqueous humor samples were collected at different time points, and CsA concentrations were measured using HPLC. The results displayed a sharp increase in CsA concentration in the aqueous humor at 4 h, reaching a peak concentration of 50.53 ng/mL at 6 h, without causing significant irritation in rabbit eyes. SLNs exhibited sustained drug release and high permeability, further enhancing drug utilization when formulated within SLNs. Shen et al. synthesized Cys-NLC as potential nanocarriers for topical ocular administration of CsA [245]. rapidly crosslinked under simulated physiological conditions. Thiolated NLCs did not cause discomfort or irritation and displayed sustained drug release in vitro. In rabbit eye experiments, Cys-NLCs were administered twice, with each dose containing 500 µg of CsA and a 90-s interval between administrations. HPLC analysis of CsA levels in various eye tissues revealed that Cys-NLCs had significantly higher CsA content than oil solutions and non-thiolated NLCs. Consequently, thiolated NLCs may represent a promising strategy for treating ocular surface disorders and anterior segment inflammatory diseases, such as uveitis. Luschmann et al. discovered that cyclosporine, enclosed in in-situ nanosuspension (INS) and a MS, demonstrated significantly higher concentrations in rabbit corneal tissue (1683 ± 430 ng_CsA_/g_cornea_ and 826 ± 163 ng _CsA_/g_cornea_) than Restasis^®^ (350 ng_CsA_/g_cornea_) and cationic emulsions (750 ng _CsA_/g_cornea_) [246]. These findings underscore the broad use and remarkable efficacy of nano-encapsulated CsA in inflammatory eye diseases.

**Fig. 6:**
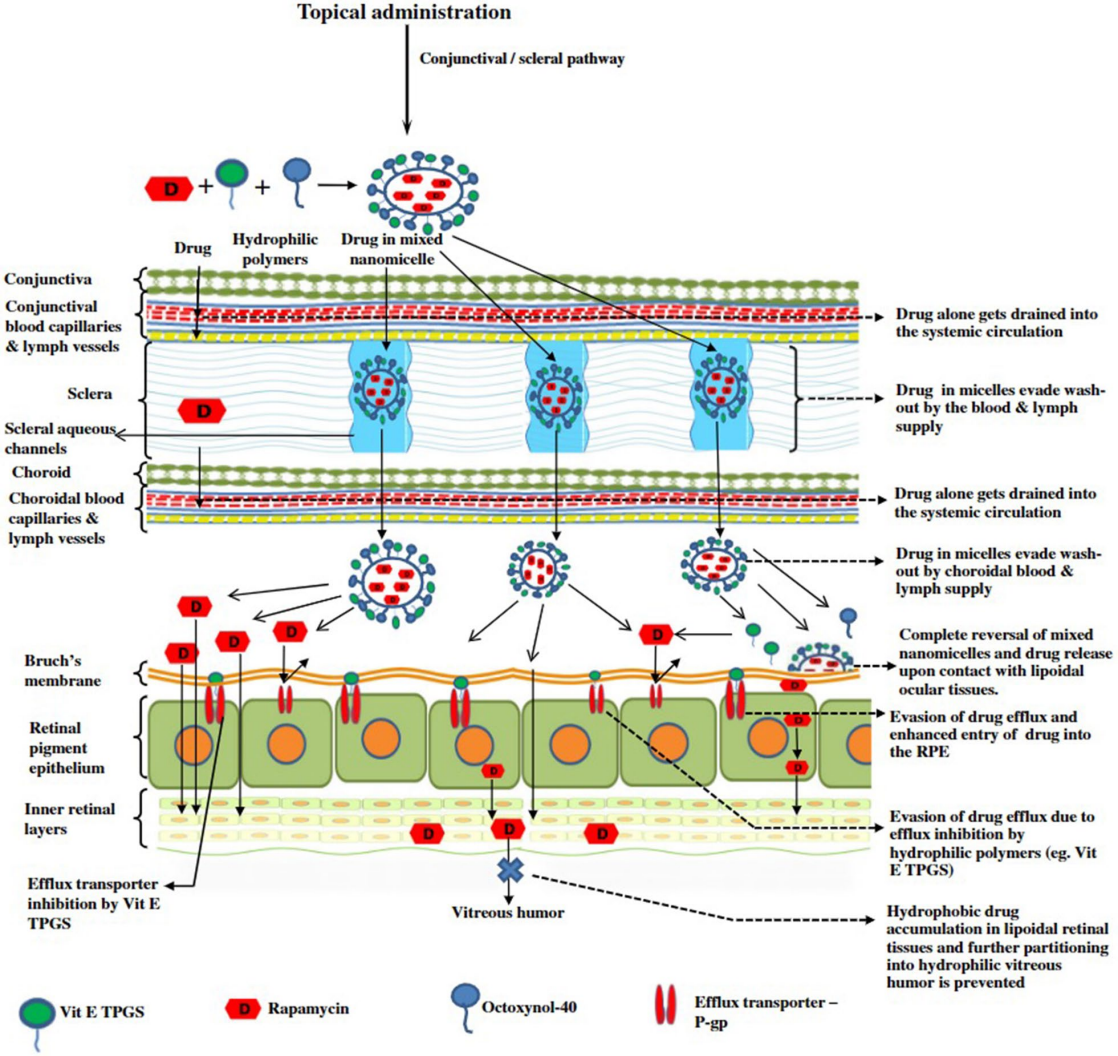
The schematic diagram demonstrates the pathway of a drug through the conjunctiva, sclera, and onward to the choroid and retinal tissue following the injection of 50 μl of 0.2% Rapamycin MNF into the conjunctival sac of rabbits. Reproduced from Ref. [247] with permission from Springer Nature

Rapamycin, a macrolide immunosuppressant generated by the bacterium Streptomyces hygroscopicus, impedes the transition of T cells from the G1 phase to the S phase of the cell cycle by inhibiting IL-2-mediated signaling pathways, thus exerting anti-inflammatory activity [260, 261]. Owing to its high hydrophobicity, orally or intravenously administered rapamycin cannot achieve effective drug concentrations for uveitis treatment. Cholkar et al. developed mixed nanomicellar formulations (MNFs) of rapamycin (0.2%) with VE tocopherol polyethylene glycol succinate and octoxynol-40 (Oc-40) as a polymeric matrix [247]. They administered 50ul of 0.2% rapamycin MNF into the rabbit conjunctival sac, and after 60 min, they collected retinal-choroidal tissue and extracted vitreous humor. The concentration of rapamycin in the tissue was 362.35 ± 56.17 ng/g tissue, while rapamycin remained undetected in the vitreous humor. These experimental outcomes suggest that the formulation exhibits drug-targeting effects in the treatment of uveitis **(**Fig. 6**)**.

**Fig. 7:**
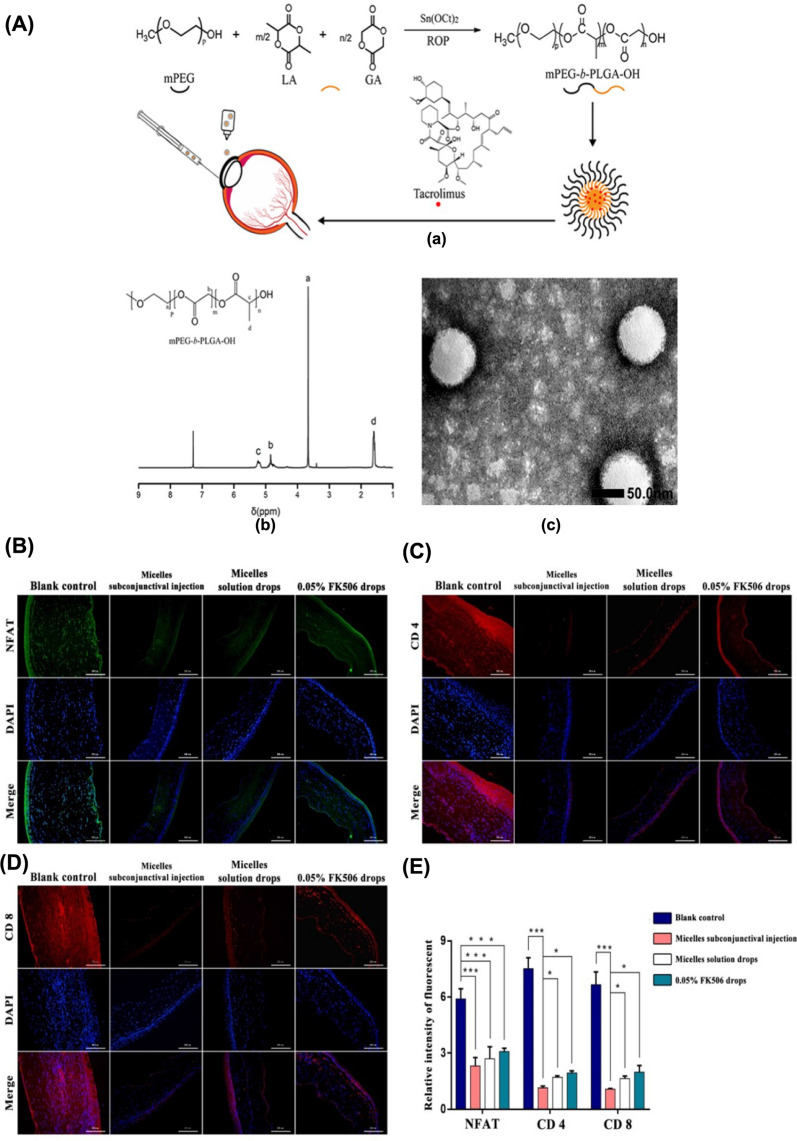
The use of Tacrolimus-loaded mPEG-b-PLGA micelles in the treatment of corneal immune rejection subsequent to allogeneic penetrating corneal transplantation in rats **A**. (a) presents a schematic diagram of the composition and ocular application of Tacrolimus-loaded mPEG-b-PLGA micelles, while (b) shows the detection of mPEG-b-PLGA by a 1H NMR spectrometer. (c) provides a scanning electron microscope (SEM) image of Tacrolimus-loaded mPEG-b-PLGA micelles with a scale bar denoting 50 nm. The immunofluorescence assay was used to observe the inhibitory effect of Tacrolimus-loaded mPEG-b-PLGA micelles (administered either via subconjunctival injection, or solution drops) on the phosphorylation of NFAT **B**, CD4 **C** and CD8 **D**, key factors in immune rejection, across various layers of corneal tissue in comparison to blank controls and standard 0.05% tacrolimus eye drops. **E** showcases a statistical graph of the percentage of fluorescence intensity. Values are represented as mean ± SD, with statistical significance denoted as *p < 0.05, **p < 0.01, ***p < 0.001. This figure is reproduced from Ref. [249] with permission from Elsevier

Analogous to rapamycin, tacrolimus (also known as TAC or FK506) demonstrates similar efficacy when incorporated with nanomaterials for treating ocular inflammation. Wu et al. devised TAC-loaded methoxy poly (ethylene glycol-block-poly (d, l)-lactic-co-glycolic acid) nanoparticles (TAC-NPs) using nanotechnology to surmount corneal transplant rejection and minimize local inflammatory responses [262]. In rats undergoing allogeneic penetrating keratoplasty, TAC-NPs enhanced the concentration of TAC in the aqueous humor and cornea compared to conventional 0.1% TAC eye drops and exhibited potent inhibitory effects on IL-2, IL-17, and VEGF expression in tissues. Liu et al. investigated FK506/NH2-PEG-b-PLA/HPMC nanomicelles [248] and TAC-loaded mPEG-b-PLGA micelles, discovering that, relative to traditional TAC eye drops, TAC incorporated into mPEG-b-PLGA micelles could diminish the expression of NFAT, CD4, and CD8 in animal tissue sections, displaying a significant inhibitory effect on immune rejection reactions following corneal allograft transplantation **(**Fig. 7**)**. The use of these formulations can reduce the risk of immune system attack on the transplanted cornea, thereby increasing the success rate of transplantation surgery and the survival rate of the transplanted cornea. This breakthrough advancement brings great hope to the field of corneal transplantation, and through ongoing research and development, these formulations are expected to be further improved and widely adopted, providing more effective immunosuppressive strategies for corneal transplant patients. Kalam et al. optimized TAC (TAC)-loaded PLGA-NPs to augment their TAC encapsulation capacity [250]. In vitro experiments using rabbit corneal tissue demonstrated that PLGA-NPs enhanced the bioavailability of TAC in the corneal, conjunctival, and aqueous humor, indicating their potential in treating ocular inflammation. Deepika et al. developed TAC gellan gum nanoparticles (TGNPs) for dry eye syndrome treatment. In a rabbit eye experiment, TGNPs exhibited prolonged drug release and elevated corneal retention within 12 h. Pharmacological studies indicated that TGNPs effectively treated DED symptoms [251]. Mayara et al. integrated TAC and MSNAPTES into silica NPs [252]. They examined the particles’ toxicity and biocompatibility in ARPE-19 and CAM models and assessed the safety of intravitreal injections using electroretinography (ERG) and rat ocular histology. No retinal, vitreous, or optic nerve lesions were detected. Moutaz et al. encapsulated TAC in a CS-based amphiphile to generate water-soluble nanoparticles (MET-TAC) [253]. One hour post-administration, TAC concentrations in the rabbit cornea and conjunctiva reached 4452 ± 2289 and 516 ± 180 ng/g of tissue, respectively. The formulation achieved effective drug concentrations in affected tissues and delivered sufficient TAC to treat moderate to severe atopic keratoconjunctivitis (AKC) and vernal keratoconjunctivitis (VKC). Rebibo et al. formulated nano-capsules (NCs) loaded with TAC, which significantly mitigated LPS-induced keratitis and experimental autoimmune uveitis (EAU) inflammatory responses [254]. The NCs inhibited the expression of inflammatory and chemotactic cytokines such as KC, MIP-2, IL-6, and GCSF in a mouse corneal inflammation model. Clinical and histological efficacy was demonstrated in a mouse (EAU) model. Sun et al. discovered that a novel in situ gel of TAC-loaded SLNs could suppress the release of inflammatory mediators from conjunctival mast cells, down-regulate IL-4 in serum, thereby inhibiting B cell antibody reactions from IgM to IgE, reducing IgE synthesis, up-regulating IFN-γ in serum, inhibiting Th2 cell proliferation and IL-4 function, suppressing the conversion of Th1 to Th2, and maintaining the dynamic balance of Th1 and Th2. Furthermore, it inhibited the expression of OVA-sIgE, IFN-γ, and IL-4 in a mouse conjunctivitis model, controlled type I allergic reactions, and treated immune-mediated conjunctivitis [255]. Collectively, these experimental studies demonstrate that biodegradable polymeric nanomaterials for drug delivery of TAC hold significant potential in enhancing clinical therapeutic effects.

In conclusion, nanomaterial-based immunosuppressive agents constitute a group of medications aimed at attenuating or inhibiting the body’s immune response. Frequently prescribed for the prevention of immune-mediated organ or tissue rejection in transplant recipients, these drugs also serve as treatment options for autoimmune diseases such as rheumatoid arthritis, lupus, and multiple sclerosis. In recent years, the exploration of alternative nanocarriers, including hydrogels, dendrimers, and micelles, for ocular drug delivery has broadened the scope of nanotechnology applications in the field of ophthalmology. Despite considerable advancements in the development of nanomaterial-based drug delivery systems for ocular conditions, further research and clinical trials are imperative to comprehensively assess their safety and effectiveness. Future investigations should prioritize the optimization of nanoparticle properties, assessment of long-term impacts, and resolution of potential challenges related to drug stability, biocompatibility, and immunogenicity.

### The delivery of TNF-α inhibitor

TNF-α inhibitors represent a group of medications designed to obstruct the function of TNF-α, a cytokine implicated in systemic inflammation and immune system regulation. Widely employed in the management of autoimmune diseases such as rheumatoid arthritis, psoriasis, and inflammatory bowel disease, these drugs include infliximab, etanercept, adalimumab, golimumab, and certolizumab pegol [263]. TNF-α is recognized as a crucial factor in the development of numerous inflammatory ocular conditions, encompassing uveitis, scleritis, and ocular surface disorders like dry eye disease [264–267]. Consequently, TNF-α inhibitors exhibit considerable potential in treating various ophthalmic ailments. Recently, nanomaterial-based drug delivery systems have demonstrated promising outcomes in addressing ophthalmic diseases using TNF-α inhibitors. Infliximab, a TNF-α inhibitor, has been proven to effectively mitigate chronic uveitis. Zhang et al. employed liposomes loaded with infliximab in an experimental autoimmune uveoretinitis rat model, administering the drug via intravitreal injections. Owing to the liposomes’ desirable biocompatibility and sustained drug release properties, a significantly enhanced reduction in inflammatory cell infiltration, diminished retinal damage, and decreased intraocular inflammation were observed in comparison to traditional infliximab, indicating potential advantages for efficient ocular tissue drug delivery [268]. This study thus highlights the potential of nanomaterial-based drug delivery systems in ophthalmology for the effective management of TNF-α mediated inflammatory conditions.

### The delivery of genes

The clinical implementation and advancement of nucleic acid therapeutics are intimately linked to effective and safe delivery systems that must accommodate the properties of genetic material and target tissues. Delivery is considered the primary obstacle in gene therapy, and it is particularly crucial to the success of corneal gene therapy in ophthalmic diseases. Presently, virus-mediated nucleic acid delivery, involving retroviruses, lentiviruses, and adenoviruses, is the most prevalent method for selecting gene therapy vectors and has been extensively employed in the investigation and treatment of ophthalmic conditions. However, immune rejection and inflammatory reactions are inescapable [269]. As a result, non-viral gene therapy, utilizing NPs as gene carriers for treating eye diseases, has gained prominence [270–273]. Commonly employed nanoscale gene carriers include SLNs [271], HA, CS [270], AuNPs [272], and magnetic NPs [274]. These carriers exhibit greater safety and reduced harm during production, while offering more convenience than viral carriers in clinical or practical applications.

Numerous studies have demonstrated that gene therapy can deliver specific anti-inflammatory factors to alleviate various types of corneal inflammation. IL-10 is an immune regulatory factor involved in antigen presentation, which can inhibit the production of pro-inflammatory cytokines IL-1, IL-6, IL-8, and TNF-α [269, 275–279], and has potent anti-inflammatory effects [280]. The plasmid encoding IL-10 was first formulated. In cell experiments, HA-SLN transfected with pUNO1-hIL10 plasmids to transfect HCE-2 cells for 72 h, IL-10 was detected in the culture medium at a concentration of 9.1 ± 0.8 ng/mL. Following local administration to wild-type and IL-10 knockout (KO) mice, it was discovered that the addition of PVA enhanced the corneal permeability of liposomes. IL-10 expression was observed in the corneal epithelium after three days of local administration of HA-SLN encoding IL-10 plasmids [281]. The use of NPs as carriers for therapeutic genetic material, delivering them to target tissues and addressing various eye diseases, has gained popularity [282, 283]. Fuente et al. compared the efficacy of a novel DNA nanocarrier coated with HA and CS [270], finding that CS-derived NPs increased alkaline phosphatase expression in a human corneal epithelial model. Plasmid DNA coated with both types of NPs could enter corneal and conjunctival epithelial cells, effectively delivering DNA. Consequently, these NPs may represent innovative strategies for gene therapy in diverse eye diseases. MUC5AC is a high-molecular-weight glycoprotein that forms a gel layer on mucous membrane surfaces, providing tissue protection. Its reduced expression is closely related to the pathogenesis of dry eye syndrome. Contreras-Ruiz et al. developed NPs carrying plasmids encoding modified MUC5AC protein (pMUC5AC) [284]. In an experimental dry eye (EDE) mouse model, tear production improved significantly after pMUC5AC-NP treatment. Fluorescent staining of lesion tissue revealed normal structure and morphology, while immunohistochemistry showed decreased CD4 or T cell infiltration and reduced inflammatory responses. MUC5AC protein expression encapsulated in nanospheres was higher in ocular surface tissues than in the control group.

Employing nanomaterials as gene carriers enables the targeting of specific genes involved in inflammation for silencing, resulting in decreased inflammation and improved disease outcomes. In summary, nanomaterial-mediated gene silencing represents a novel and promising approach for the treatment of inflammatory eye diseases, with the potential to enhance patient outcomes.

### The delivery of natural products with anti-inflammatory properties

Natural substances possessing anti-inflammatory properties have long been employed in treating various inflammatory conditions. Often derived from plants and fungi, these substances have demonstrated efficacy in reducing inflammation and promoting overall health. Nanomaterial-based drug delivery systems have emerged as a promising approach to enhance the therapeutic potential of these anti-inflammatory natural substances in ophthalmic diseases **(**Table 6**)**. Such systems offer numerous advantages, including improved solubility, targeted delivery, enhanced bioavailability, and controlled release of the natural products [285, 286].

**Table 6:** The application of nanomaterials in combination with natural anti-inflammatory agents in the treatment of ocular diseases

Natural products	Nanomaterials	Size (nm)	Production method	Cells (in vitro)	Animals (in vivo)	Administration route	Characteristics and effects	Refs.
Resveratrol	AuNPs	20	Ecofriendly synthetic method	–	STZ-induced diabetic rats	Oral	Resveratrol-loaded AuNPs exhibited decreased BRB permeability, increased retinal expression of PEDF, decreased VEGF-1 expression, and reduced retinal mRNA expressions of VEGF-1, TNF-α, MCP-1, ICAM-1, and IL-6, and IL-1β	[287]
Myr	PVCL-PVA-PEG polymeric nanomicelles	60.72 ± 1.09	Thin-film hydration method	HCECs	Rabbit and mouse	Eye drops	Myr-loaded PVCL-PVA-PEG polymeric nanomicelles exhibited enhanced aqueous solubility and chemical stability, superior storage stability, favorable cellular tolerance, improved cellular uptake and corneal permeability, and improved antioxidant and anti-inflammatory effect	[288]
CUR	PVCL-PVA-PEG nanomicelles	50.1 ± 1.0	Solvent evaporation and film hydration method	HCECs	Rabbit and mouse	Eye drops	CUR-loaded PVCL-PVA-PEG nanomicelles exhibited physical stability, favorable cellular tolerance, high corneal permeability, and antioxidant and anti-inflammatory effect	[289]
CUR	PLGA-GA_2_ polyester NPs	250	Single emulsification method	HCECs	Homologous lens protein-induced uveitis in beagles	oral	CUR-loaded PLGA-GA_2_ polyester NPs exhibited no observed cytotoxicity, attainment of effective drug concentration in aqueous humor, and inhibition of intraocular inflammation	[186]
OA and UA	Poloxamer 188 polymeric NPs	< 225	Solvent displacement method	Isolated corneas of rabbits and HET-CAM test	SA-induced ocular inflammation in rabbits	Eye drops	OA and UA-loaded NPs exhibited excellent corneal permeability, remarkable safety, and potent anti-inflammatory effects	[290]

*STZ* streptozotocin; *Myr* myricetin; *CUR* curcumin; *PVCL-PVA-PEG* polyvinyl caprolactam-polyvinyl acetate-polyethylene glycol; *GA* gambogic acid; *OA* oleanolic acid; *UA* ursolic acid

**Fig. 8:**
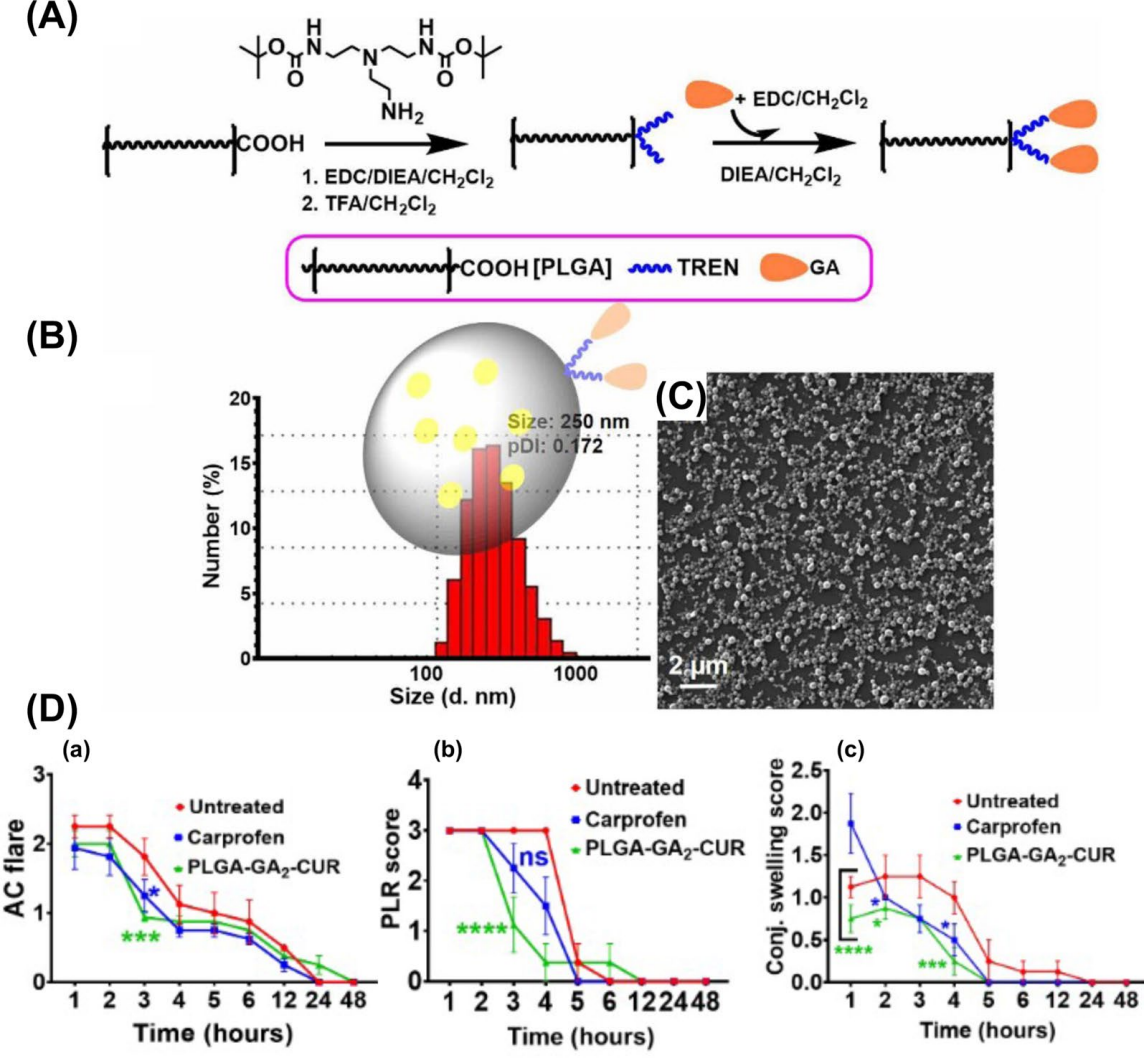
The synthesis of PLGA-GA_2_-CUR and its resultant therapeutic impact on a beagle uveitis model. **A** The schematic diagram illustrates the composition of PLGA-GA_2_-CUR. **B** A dynamic size distribution of light scattering describes the model particle of PLGA-GA_2_-CUR. **C** SEM provides microstructure images of PLGA-GA_2_-CUR. **D** The anti-inflammatory effect of topical PLGA-GA_2_-CUR is demonstrated in a canine model of acute endophthalmitis. Following an intraocular injection of lens protein at t = 0 h, the semiquantitative preclinical ocular toxicology scoring (SPOTS) was employed, incorporating scores for aqueous flare (a), pupillary light reflex (b), and conjunctival swelling (c). Local administration of PLA-GA_2_-CUR showed statistical significance when compared to topical prednisolone acetate (PA) and untreated controls, as assessed by two-way ANOVA. Statistical significance is denoted as *p < 0.05, **p < 0.01, ***p < 0.001, and ****p < 0.0001. This figure is reproduced from Ref. [186] with permission from the American Association for the Advancement of Science

Resveratrol, a bioactive component found in grape juice and Polygonum cuspidatum, had demonstrated efficacy in reducing BRB permeability and lowering TNF-α, MCP-1, IL-6, and IL-1β mRNA expression in the retina by inhibiting the phosphorylation of NF-κB and ERK in STZ-induced DR in rats when delivered via gold nanoparticle-coated resveratrol nanopreparations [287]. The anti-angiogenic effect of these nanopreparations suggests potential therapeutic value in inflammatory neovascular eye diseases. Myricetin (Myr) is a natural flavonol compound utilized in treating organism lesions. Its anti-inflammatory properties are beneficial for degenerative and inflammatory eye diseases, such as dry eye syndrome and chronic anterior uveitis [291–294]. To address Myr’s poor water solubility and low stability, researchers encapsulated Myr in PVCL-PVA-PEG polymer micelles, which resulted in increased water solubility and stability. In vivo anti-inflammatory experiments demonstrated a dose-dependent anti-inflammatory effect with 4 mg/ml Myr micelle eye drops exhibiting strong anti-inflammatory effects, comparable to pranoprofen eye drops [294]. Curcumin (CUR), a bioactive component found in turmeric, has been extensively researched and employed in the treatment of ocular diseases due to its capacity to inhibit corneal epithelial cell neovascularization, lens epithelial cell proliferation, protect retinal ganglion cells, and suppress choroidal neovascularization [295, 296], as well as its anti-inflammatory and neuroprotective properties [297]. Li et al. encapsulated CUR within PVCL-PVA-PEG polymer micelles, observing that combining CUR with nanomaterials significantly enhanced corneal bioavailability and ocular tolerance [288]. Moreover, 4.5 mg/ml nanomicelle CUR demonstrated anti-inflammatory effects comparable to pranoprofen. In another study, Ganugula et al. combined CUR with PLGA and encapsulated the mixture in double-headed polyester NPs [186]. Oral administration of PLGA-GA_2_-CUR enabled the detection of CUR content in aqueous humor, suggesting that PLGA-GA_2_-CUR can traverse the BRB. In an acute anterior uveitis beagle model, oral administration of PLGA-GA_2_-CUR significantly ameliorated aqueous humor inflammation and intraocular edema **(**Fig. 8**)**. Pentacyclic triterpenoids constitute a diverse and widespread class of natural compounds with a wealth of resources. Numerous studies have highlighted their wide-ranging pharmacological and biologically significant activities, particularly in the domains of anti-inflammatory and immune regulation, garnering significant interest. Oleanolic acid (OA) and ursolic acid (UA) are anti-inflammatory compounds extracted from the leaves of *Thymus broussonetii* and *Thymus willdenowii*, which are part of the Lamiaceae family and are rich in these compounds [298]. These triterpenoids impede inflammation progression by inhibiting cyclooxygenase (COX) and PLA2 activity, blocking the release of cytokines, histamine, and serotonin, and interacting with serine/threonine kinases [299]. Helen et al. optimized PLGA-NPs loaded with OA and UA using a 2^3^ + star CCRD [290]. The study revealed that OA/UA-loaded nanomaterials (NM OA/UA NPs) exhibited excellent permeability and safety in corneal tissue during in vitro rabbit corneal tissue penetration experiments and HET-CAM studies. Additionally, NM OA/UA NPs maintained a higher concentration of drugs in the corneal tissue compared to the standard mixture (SM) post-administration. The anti-inflammatory effects of SM or NM OA/UA NPs were assessed 30 min after administration, followed by the application of SA solution (SAS) and quantification of intraocular inflammation using the Draize test. The results indicated that NM OA/UA NPs displayed superior anti-inflammatory activity compared to the simple OA/UA mixture.

In conclusion, the integration of natural products with nanomaterial-based drug delivery systems has the potential to amplify their anti-inflammatory properties, which could pave the way for the development of more effective and targeted therapies for addressing inflammation and associated diseases.

## Other pharmacological activities of nanomaterials in ophthalmology diseases

**Fig. 9:**
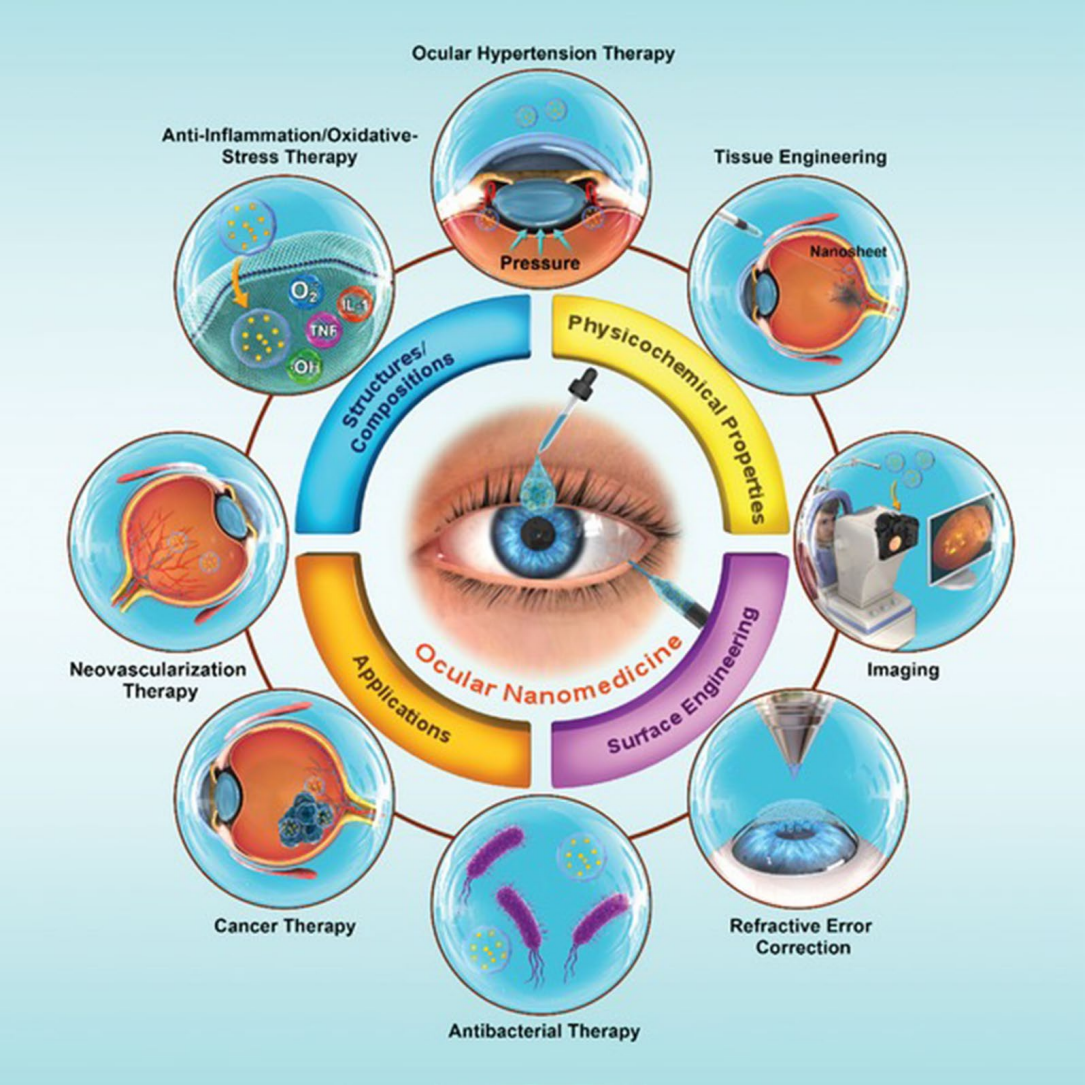
A schematic illustration of the diverse aspects of nanomaterial application in ocular biomedicine. This figure is reproduced from Ref [344]. with permission from John Wiley and Sons

The applications of nanomaterials in ophthalmology reach far beyond their well-established anti-inflammatory effects and drug delivery capabilities. These minuscule particles offer a range of multifunctional properties that can be harnessed for diverse therapeutic purposes, including antioxidation, anticancer, tissue engineering and regeneration, ocular imaging, and correction of refractive errors. In this section, we will discuss the potential benefits of nanomaterials as well as the challenges that must be addressed to successfully implement them in clinical practice **(**Fig. 9**)**.

### Antioxidation

**Fig. 10:**
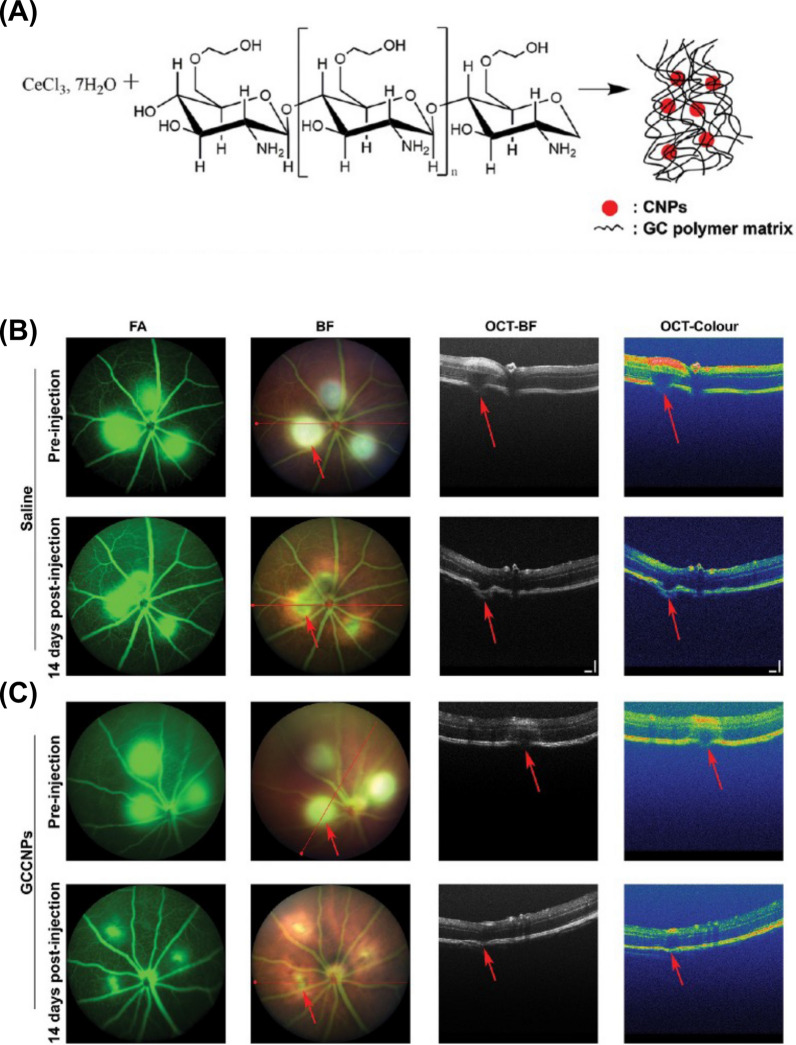
The inhibitory effect of GCCNPs on neovascularization in a laser-induced choroidal neovascularization mouse model.** A** A schematic diagram depicting the synthesis, structure, and morphology of GCCNP is presented. The protective role of GCCNP on laser-induced lesions is highlighted in a mouse model of choroidal neovascularization. The control group received a vitreal injection of saline **B**, while the experimental group was administered a vitreal injection of GCCNPs **C**. Observations of the repair of damaged fundus vessels by the drug were conducted both prior to and 14 days post-injection using fundus fluorescein angiography, plain fundus photography, and OCT. Laser-induced lesion sites are indicated by red arrows. The representations include fluorescein angiography (FA) and bright field (BF) images. This figure is reproduced from Ref. [305] with permission from the American Chemical Society

Oxidative stress has been implicated in various ocular diseases, such as AMD [226] and cataractss [300]. NPs can be engineered to neutralize free radicals and inhibit oxidative damage, potentially preventing or decelerating the progression of these diseases. Wet AMD is a choroidal neovascularization disease that originates from endothelial cell dysfunction. Research indicates that oxidative stress is involved in the development of AMD and is positively correlated with pathological vascular lesions [301–303]. Regrettably, there is a scarcity of effective drugs based on antioxidant damage therapy for treating AMD, making the development of drugs capable of effectively clearing ROS to treat wet AMD a critical endeavor. Nanomedicine has facilitated the development of novel ROS-clearing techniques, employing a variety of functional nanomaterials to address ROS-related diseases [304]. Experimental evidence demonstrates that biocompatible and stable nanoceria formulations, such as glycol CS-coated ceria nanoparticles (GCCNPs), exhibit potent antioxidant activity. In vitro experiments reveal that GCCNPs could suppress the expression of VEGF in ARPE19 cells and HUVECs induced by H_2_O_2_, inhibit the vascular formation and migration of HUVECs induced by H_2_O_2_, and inhibit the oxidative reaction product 4-HNE and the chemokine stromal-derived factor-1 (SDF-1) and its receptor CXCR4 in laser-induced choroidal neovascularization C57 model mice following intravitreal injection. GCCNPs accumulated more at the site of laser-induced CNV injury (RPE layer) [305] **(**Fig. 10**).** Many drug-loaded nanomaterials also exhibit strong antioxidant properties, in addition to the nanomaterials themselves. For example, eye preparations based on dipotassium glycyrrhizinate (DG)-loaded nanomicelles carrying thymol (THY) display enhanced Fe^3+^ reduction activity in FRAP tests compared to free THY [306]. In vivo and in vitro experiments show that nanomaterials effectively mitigate oxidative stress and suppress inflammatory reactions in ocular lesions, offering advantages over corresponding free drugs by producing more favorable outcomes and preserving drug efficacy stability during transportation and storage. As a result, the antioxidant capacity of nanomaterials warrants further investigation in the treatment of ocular diseases, as an efficacious and safe therapy for various conditions associated with oxidative stress.

### Anticancer

Ocular neoplasms encompass tumors of the eyelids, conjunctiva, various layers of the eyeball, and ocular appendages. These neoplasms are classified as benign or malignant based on their pathological characteristics, each displaying distinct features and age of onset within the population. Retinoblastoma (RB) represents the most common intraocular malignancy affecting children, originating from mutations or deletions in the RB1 tumor suppressor gene within developing retinal cells—an autosomal dominant inherited disorder [307]. The typical clinical manifestation in affected children is a yellow-white glow within the pupillary region, accompanied by reduced vision, ocular discomfort, and inflammation. Currently, numerous treatment modalities for RB exist, including intravenous and arterial chemotherapy, external radiation, cryotherapy, and enucleation [308]. However, repeated administration of these treatments may induce systemic and cellular toxicity, tumor dissemination, infection, and additional complications. Recent advancements in nanotechnology have facilitated progress in RB treatment. NPs have been utilized to reduce chemotherapy toxicity and overcome challenges in drug transport across the ocular barrier. NPs have been utilized to reduce chemotherapy toxicity and overcome challenges in drug transport across the ocular barrier [309, 310].

**Fig. 11:**
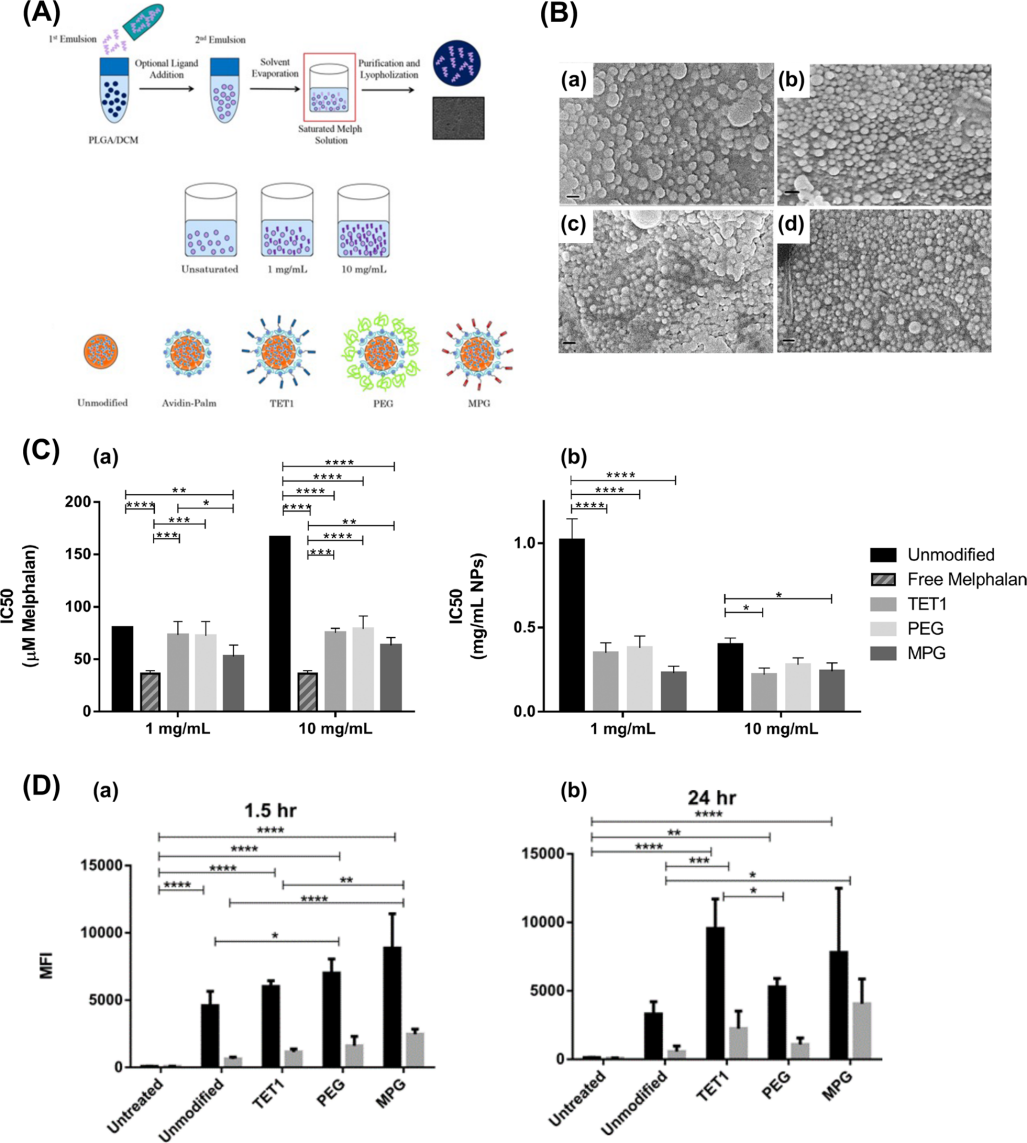
The therapeutic impact of surface-modified melphalan nanoparticles (NPs) on retinoblastoma (RB). **A** A schematic depiction of the preparation and concentration of melphalan is provided. **B** Structural schematic diagrams and scanning electron microscope (SEM) images of four distinct NP preparations are depicted: Surface-unmodified PLGA NPs loaded with melphalan (a), surface-modified PLGA NPs loaded with melphalan by TET1 (b), surface-modified PLGA NPs loaded with melphalan by PEG (c), and surface-modified PLGA NPs loaded with melphalan by MPG (d), scale bar = 200 nm. **C** In an in vitro cellular assay, four different NP formulations in 1 mg/mL and 10 mg/mL melphalan configurations were used to treat Y79 cells for 24 h to observe their cytotoxic effects. MPG NPs were identified as the most effective treatment group. IC50 values for TET1, PEG, and unmodified NPs were higher than those for free melphalan, while MPG NPs demonstrated statistically similar efficacy to free melphalan. IC50 values are displayed as mean ± SD; statistical significance is indicated by *P ≤ 0.05, **P ≤ 0.01, ***P ≤ 0.001, ****P ≤ 0.0001. **D** The influence of surface modification of NPs on Y79 cell binding (black) and internalization (gray) was assessed using flow cytometry. Surface-modified NPs showed increased cell binding and internalization compared to unmodified NPs at 1.5 h (a) and 24 h (b). Data is represented as mean ± SD; statistical significance is indicated by *P ≤ 0.05, **P ≤ 0.01, ***P ≤ 0.001, ****P ≤ 0.0001. This figure is reproduced from Ref. [345] with permission from the Association for Research in Vision and Ophthalmology Inc

Melphalan is the primary chemotherapeutic agent used for arterial RB treatment; however, repeated injections and anesthesia may prove harmful. Lee et al. employed a double emulsion synthesis technique to encapsulate melphalan within PLGA-NPs, observing increased efficacy and an extended interval between successive injections [310]. MicroRNA plays a crucial role in regulating the development of various diseases, including RB. MiRNA-181a was downregulated in RB and other cancerous cells [311, 312]. Combining microRNA and chemotherapeutic agents may enhance treatment effectiveness by modulating the chemical susceptibility of malignant cells [313–315]. Tabatabaei et al. developed a lipid nanoparticle delivery system for melphalan and miRNA-181a, resulting in superior transfection efficiency and reduced toxicity in Y79 cells and RB heterotopic transplant rat models [316]** (**Fig. 11**)**.

Targeted therapy has attracted considerable attention in cancer treatment due to its increased specificity and efficacy. Sugar receptors are overexpressed in retinoblastoma, and Rutika et al. designed an innovative sugar receptor-targeted drug delivery system for Etoposide (ETP) [317]. ETP-PLGA-NPs were synthesized using the solvent displacement method, demonstrating sustained drug release for 32 h. In Y79 cells with an excess of sugar receptors, ETP-PLGA-NP uptaked exceeded that of non-binding ENP, and the NPs exhibited higher cancer cell apoptosis rates compared to pure ETP.

Metal NPs possess anti-inflammatory properties and unique advantages in RB treatment. AgNPs derived from pure algal aqueous extract solutions were characterized, exhibiting significant cytotoxicity in Y79 cell lines. Incorporating polysaccharides into the synthesized AgNPs reduced their toxicity in Y79 cells and enhanced free radical elimination [317]. While the formulation has undergone toxicological validation through in vitro cell experiments, it is well-known that in vivo animal studies provide a better simulation of the real biological environment and offer more comprehensive data and assessments. Therefore, in future research, it is necessary to conduct further in vivo animal experiments to gain a better understanding of the formulation’s performance in living systems and evaluate its potential effects in treatment or other applications.In contrast to conventional cancer therapies, nanomaterials-mediated drug delivery offers superior efficacy, decreased toxicity, and ligand-specific targeting, thus managing cellular toxicity and improving cost-effectiveness. The emerging trend of multifunctional and biocompatible ligands is strategically improving the treatment and diagnosis of RB, signaling a new era in overcoming challenges associated with traditional therapy.

### Tissue engineering and regeneration

Regenerative medicine presents a renewed prospect for restoring aged and diseased organs, with notable progress in ocular medicine, particularly concerning corneal, crystalline lens, and retinal disorders, facilitating the recuperation of compromised tissue functionality. Nanomaterials provide an optimal scaffold for corneal tissue regeneration, exhibiting stability. Iriczalli et al. had enhanced traditional nanoscaffolds by incorporating natural wool keratin fibers into polycaprolactone nanoscaffolds. A composite scaffold with superior light transmittance and reduced fiber keratin degradation was created when mixed in a 1:1 ratio. MSCs were cultivated on these scaffolds, sustaining growth and metabolism for up to two weeks [18]. Limbal epithelial stem cells proliferate and differentiate during corneal injury, repairing damaged tissue. Exogenous SDF-1α augments stem cell proliferation, chemotaxis, and migration, increasing the expression of differentiation-related genes in vitro in LESC and MSCs. Tang et al. found that in a rat alkali injury model, treatment with SDF-1α-loaded thermosensitive CS-gelatin hydrogel (CHI hydrogel) for 13 days resulted in the proliferation, thickening, and orderly arrangement of corneal epithelial cells, as observed via transmission electron microscopy. This mechanism may involve the secretion of growth factors in the SDF-1/CXCR4 chemotactic axis to regulate cell proliferation [318]. Subsequently, the researchers discovered that combining MSC-derived exosomes with thermosensitive CHI hydrogel could downregulate the expression of type I and V collagen. The exosome-contained miR-432-5p inhibits Translocation-associated Membrane Protein 2 (TRAM2), preventing extracellular matrix deposition and effectively promoting the repair of damaged corneal epithelium and stromal layer, reducing scar formation and accelerating the healing process [319].

**Fig. 12:**
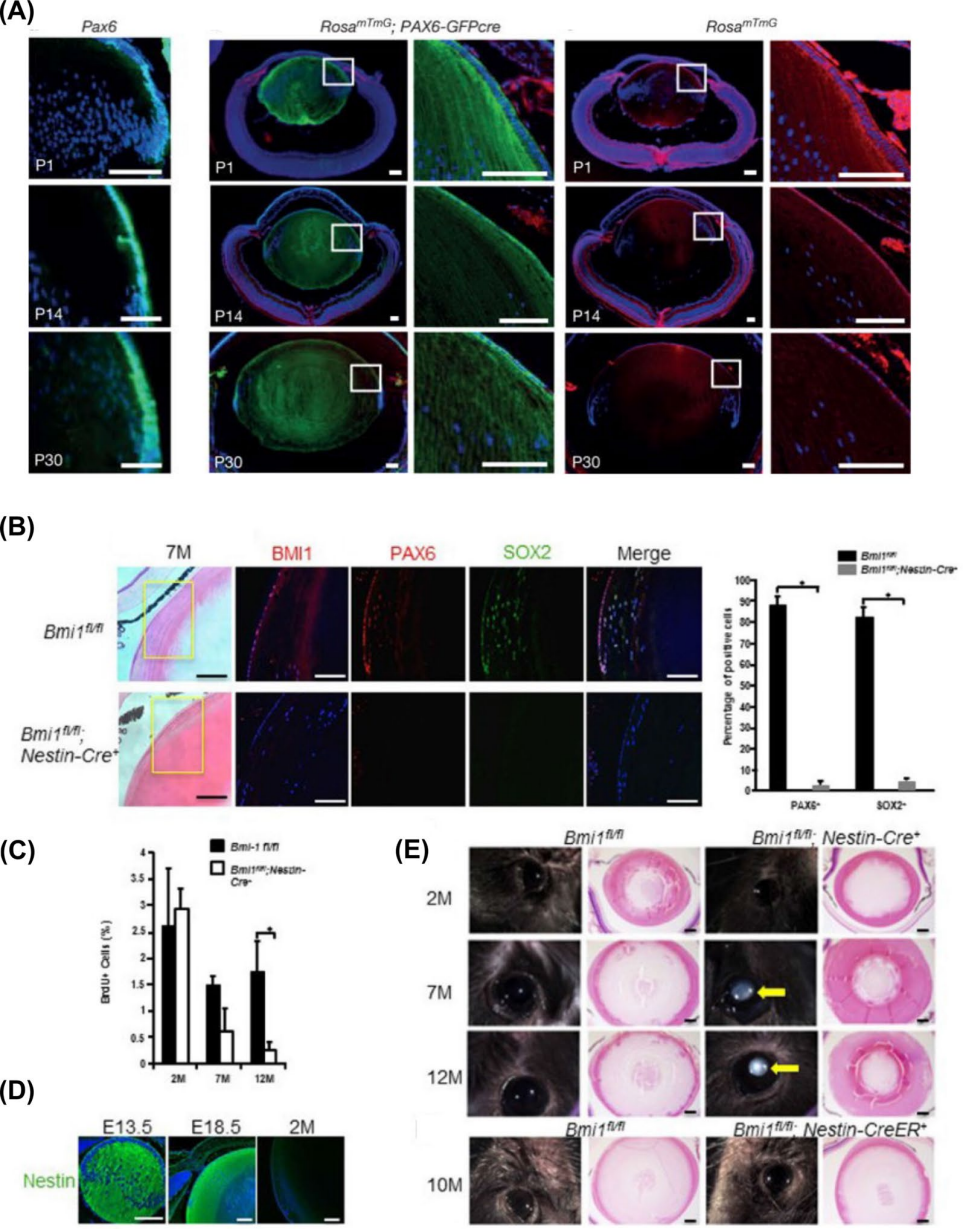
The pivotal roles of *Pax6*, *Bmi1*, and *Ki67* in ocular development and lens-induced regeneration. **A** This panel illustrates the role of Pax6 in eye and lens development. In progeny derived from crossing the *ROSA*^*mTmG*^ membrane-bound GFP reporter strain with (*P0-3.9-GFPcre*) mice, robust GFP expression was noted in lens epithelial cells (LECs) under fluorescence microscopy, indicating that Pax6 LECs from embryonic or adult lenses contribute to the regeneration of mouse lens fiber cells. **B** The part shows that deletion of Bmi-1 results in diminished Pax6 + and Sox2 + expression in LECs. The vertical axis represents the percentage of Pax6 + and SOX2 + cells. Data are presented as mean ± SD; statistical significance is denoted by *P < 0.001. The part **C** reveals that the absence of Bmi1 leads to a decrease in LEC proliferation. The proportion of BrdU + LECs was calculated for each eye at 2 m, 7 m, and 12 m. Statistical significance was determined via a two-tailed Student’s t-test; *P < 0.05. Part **D** displays Nestin (green) staining images of wild-type mice at E13.5, E18.5, and 2 months of age, as observed through fluorescence microscopy. Part **E** depicts the histological examination (HE staining) of eyeballs from 2 m, 7 m, and 12-month-old Bmi1fl/fl control mice and Nestin-cre; Bmi1fl/fl mice, to observe the development of cataracts. After using Nestn-creER to delete Bmi-1 in 6-week-old mice and following 10 months of tamoxifen treatment, the HE morphology of mouse eyes showed no cataract phenotype. All scale bars equal 100 μm. Reproduced from Ref. [346] with permission from Springer Nature

**Fig. 13:**
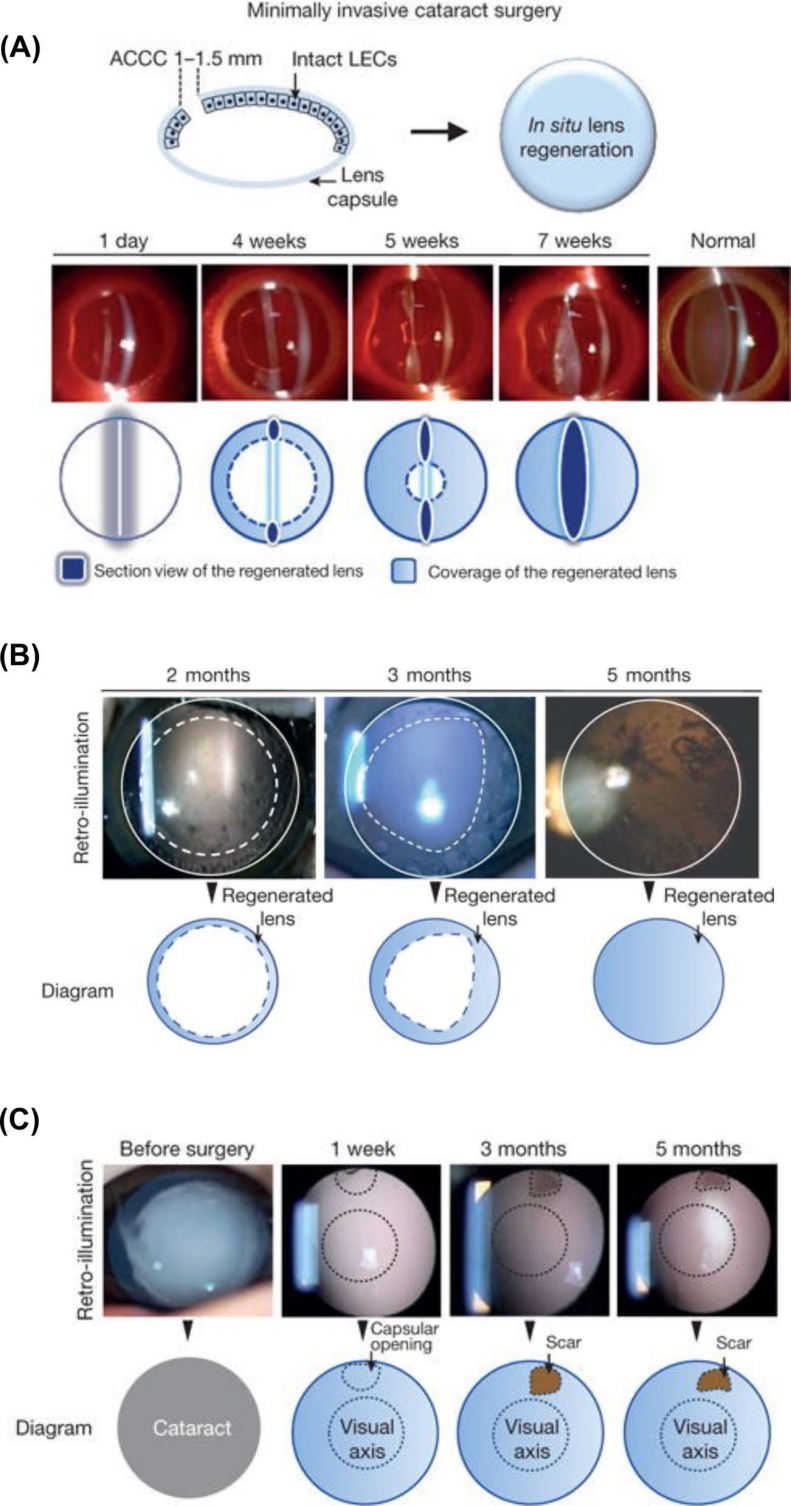
A novel minimally invasive surgical technique for promoting lens regeneration. **A** A minimally invasive ocular surgery conducted on a rabbit eye, employing a capsulorhexis size of 1–1.5 mm. The procedure targets a 1.2 mm2 region surrounding the lens, with photographic evidence of lens regeneration observed via a slit-lamp microscope from day 1 to 4 weeks post-surgery. **B** A similar minimally invasive ocular operation was executed in a macaque model. Slit lamp microscopy demonstrates the regenerated lens tissue expanding from the periphery towards the lens center between 2–5 months post-surgery. Direct illumination reveals a translucent visual axis. **C** The minimally invasive procedure was also performed on infants with congenital cataracts. Lens regeneration was observed from week 1 to 5 months post-surgery, with almost all eyes (95.8%) attaining visual axis transparency. The surgical incision remained peripheral, and the anterior capsule wound scar tissue kept away from the visual axis was less than 1.5 mm in diameter. This novel surgical approach significantly mitigated visual axis opacity compared to the current standard surgical approach. Reproduced from Ref. [346] with permission from Springer Nature

The enigma of lens regeneration in the eye has persisted throughout history, in addition to corneal stem cell regenerative therapy. Lin et al. investigated the central roles of Pax6 and Bmi1 in eye development and lens induction. In mice obtained from breeding the *ROSA*^*mTmG*^ membrane-bound GFP reporter strain with (*P0-3.9-GFPcre*) mice, robust GFP expression was observed in lens epithelial cells (LECs) under fluorescence microscopy. This indicated that Pax6 LECs derived from embryonic or adult lenses contributed to mouse lens fiber cell regeneration. In *Pax6P0-3.9-GFPcre* mouse lens anterior capsule, Pax6^+^ (GFP-positive) LECs exhibited higher *Bmi1*, *Sox2*, and *Ki67* expression levels compared to Pax6^−^ (GFP-negative) LECs **(**Fig. 12**)**. Furthermore, the researchers devised an innovative capsulorhexis method, tested in rabbits, 1–3-month-old macaques, and children under two years old with congenital cataracts, resulting in lens regeneration and exceptional visual axis transparency. This groundbreaking approach minimizes wound size, mitigates anterior capsule damage during capsulorhexis, and shifts the capsular opening from the central visual axis to the periphery, substantially preserving lens epithelial cells and promoting lens regeneration [17] **(**Fig. 13**)**. In both congenital and age-related cataract patients, the amalgamation of enhanced surgical techniques, the injection of stem cells and LEC growth-promoting components, and the employment of nanomaterial scaffolds to expedite LEC proliferation may potentially maximize lens regeneration.

The retina consists of blood vessels, nerves, and various photoreceptor cells, maintaining a diverse array of visual functions in animals. Retinal photoreceptors, including rods and cones, are responsible for detecting dim and bright light stimuli, respectively. When these cells become impaired, visual disturbances arise. Glucocorticoids are among the most commonly prescribed medications for retinal diseases, with the primary treatment method being intravitreal injections, delivering drugs directly into the vitreous to exert anti-inflammatory, immunosuppressive, and vasoconstrictive effects [320–322]. Wang et al. optimized this formulation by synthesizing nanoscale zirconium-porphyrin metal–organic frameworks (NPMOF) to encapsulate methylprednisolone (MPS) [16]. Injecting this drug into the vitreous cavity of zebrafish with light-induced retinal photoreceptor damage promoted the proliferation of cone cells, rod cells, and Muller cells, thereby enhancing retinal visual function regeneration. Although there are differences between zebrafish eyes and human eyes, the importance of zebrafish as an experimental animal model in ophthalmic research cannot be overlooked. The study of zebrafish retina provides valuable information and a platform for the development of treatments for ocular diseases in humans, thus driving forward new therapeutic strategies and drug development. Kang et al. combined injectable tauroursodeoxycholic acid and CUR with alginate to form a composite nanoscale hydrogel, which demonstrated increased adhesion to diseased tissue in vivo. Moreover, they found a 41% and 23% increase in the proliferation rate of RPE cells compared to the pure alginate group [323, 324].

In summary, tissue engineering and regeneration hold immense potential for the development of innovative therapies for various ophthalmic diseases. Although significant challenges remain, including the need for long-term safety and efficacy studies, these approaches offer a potential solution to the limitations of conventional treatments and may provide hope for patients with currently incurable eye diseases.

### Ocular imaging

Due to the distinctive architecture of the eye and the heterogeneous composition of ocular tissues, nanotechnology has made considerable progress in detecting various ocular diseases and enhancing imaging techniques. The exceptional functions and potential applications in biology and medicine render nanomaterials indispensable for augmenting disease detection in ocular imaging systems, thus facilitating clinical diagnoses.

AuNPs provide numerous advantages in ocular applications compared to traditional diagnostics [325]. Fu et al. found that femtosecond laser-prepared AuNPs, coated with polyethylene glycol, synthesized PEG-AuNPs (20.0 ± 1.5 nm), function as excellent contrast agents for photoacoustic microscopy (PAM) and OCT [326]. In vitro bovine retinal endothelial cells and in vivo rabbit experiments demonstrated no significant cytotoxicity or multi-organ toxicity. Simultaneously, the detection of blood vessels in the retina and choroid was enhanced, increasing by 82% in PAM and 45% in OCT. Subsequently, the research team coupled AuNPs with arginine-glycine-aspartic acid (RGD) peptides (CGNP clusters-RGD), synthesizing a contrast agent with a red-shifted peak wavelength of 650 nm. In a rabbit choroidal neovascularization model, following auricular vein injection of CGNP clusters-RGD, retinal examination using PAM and OCT revealed signal intensity increases of 1700% and 176% [14], respectively. This evidence provides improved clarity for subretinal neovascularization, assisting disease diagnosis and treatment plan formulation. As a high-quality contrast agent, AuNPs can also contribute to the development and application of ocular detection imaging systems. Maryse pioneered a novel OCT scanning method—Photothermal OCT (PT-OCT) [327], addressing the limitations of traditional methods, which exhibit poor detection specificity with contrast agents in a scattering background [328]. Utilizing retinal melanin as an endogenous detection material and intravenously injected gold nanorods as exogenous detection material, PT-OCT observes the photothermal effects on endogenous and exogenous substances in the retina, effectively adding a new source of contrast for structural OCT and marking a new era in ocular OCT examinations.

In conclusion, ocular imaging techniques play a vital role in understanding the applications and potential of nanomaterials in ophthalmology. As research in this area advances, it is anticipated that the utilization of nanomaterials will lead to improved diagnosis, treatment, and management of various ocular diseases.

### Vision correction

With societal progression and technological advancement, myopia prevalence has escalated among populations, including adolescents and working adults. Myopia arises from a combination of genetic and environmental factors, typified by increased axial length and thinning of the sclera at the eye’s posterior pole. Complications linked with myopia, such as vitreous opacities, retinal detachment, and macular degeneration, follow. As a challenging condition to treat that significantly affects patients’ daily lives and work, no effective pharmaceutical therapy for myopia currently exists. While myopia treatments continue to develop, existing methods primarily concentrate on rectifying the condition.

Myopia correction techniques encompass eyeglasses, contact lenses (CLs), orthokeratology lenses, corneal laser surgery, and implantable collamer lens (ICL) surgery. Eyeglasses are the primary choice, while CLs are also widely employed, and surgical correction may be considered in specific cases. The market is saturated with a diverse range of CLs, composed of various materials and exhibiting unique physical properties, resulting in different patient experiences. The application of nanomaterials in vision correction is a swiftly advancing field that holds tremendous potential for enhancing the treatment of various vision issues. Researchers have been exploring inventive methods to integrate nanomaterials into CLs, intraocular lenses, and even artificial retina development. These advancements aim to provide more effective and less invasive solutions for common vision problems such as myopia, hyperopia, presbyopia, and astigmatism, as well as more severe conditions like cataracts and macular degeneration. To further minimize spherical and chromatic aberrations, Lina et al. investigated the beneficial role of nanomaterials in optical refraction [329]. Titanium dioxide nanoparticles (TiO_2_ NPs) are a remarkable material for augmenting high refractive index (RI). Lina et al. synthesized TiO_2_ NPs using the sol–gel method and polymethyl methacrylate (PMMA) via free-radical polymerization, combining them in specific ratios to create PMMA-TiO_2_ polymer CLs. The researchers examined the performance parameter changes in ocular aberrations under polychromatic light sources, with and without the addition of TiO_2_ in CLs. PMMA-TiO_2_ CLs exhibited a high RI value of approximately 1.615, low dispersion (νd = 31), and high transparency in the visible region (T > 95%). The retinal image sharpness (spatial frequency value) and contrast (MTF value) experiments revealed that CLs with TiO_2_ demonstrated higher image contrast at low frequencies (less than 20 cycles/mm) and achieved optimal corrected visual acuity at 0.01 PMMA-TiO_2_ CL. This not only reduced spherical, coma, and astigmatism aberrations but also enhanced visual image quality. However, the evaluation of NPs’ toxicity is crucial for the development of new products, which can ensure that the addition of TiO_2_ NPs in contact lenses does not have any negative impact on ocular health, thereby ensuring the reliability and usability of the new product. Although the application of nanomaterials in vision correction remains a developing field, the potential benefits are substantial. As research progresses, we can anticipate more effective and less invasive solutions for a broad range of vision problems, improving the quality of life for millions of people worldwide.

## Safety and toxicity of nanomaterials in ophthalmology diseases

**Fig. 14:**
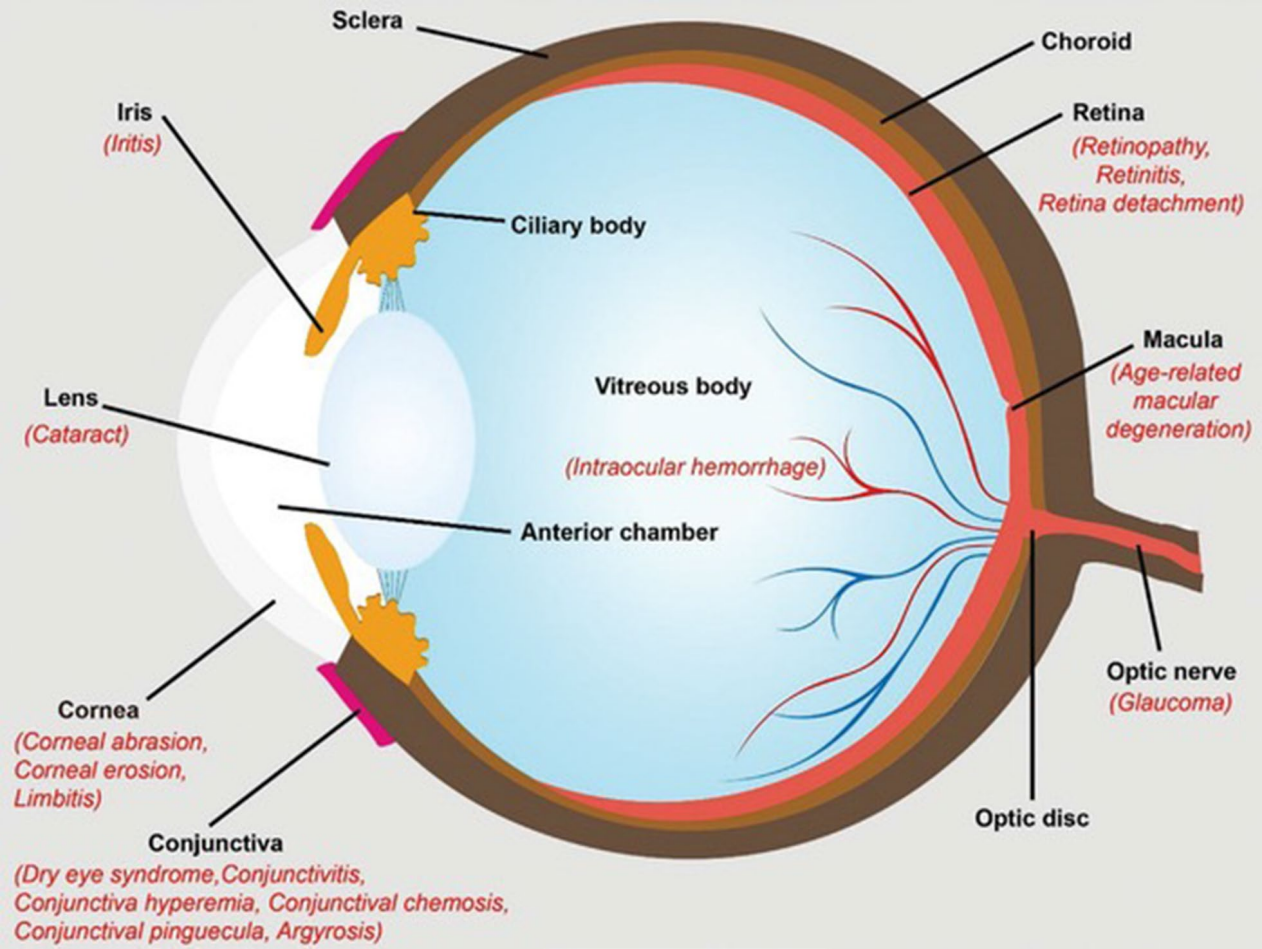
Ocular pathologies induced by the improper utilization of nanomaterials. The image showcases irritation and toxic responses elicited by nanomaterials interacting with the eye surface (cornea, conjunctiva), intraocular structures (e.g., iris, ciliary body, choroid, and lens), or various regions of the retina, macula, and optic nerve. Reproduced from Ref. [347] with permission from John Wiley and Sons

In recent years, the employment of nanomaterials has emerged as a promising strategy for addressing and diagnosing a wide array of ophthalmic disorders. Due to their unique physicochemical properties and enhanced bioavailability, these materials hold tremendous potential to revolutionize ocular therapeutics. However, alongside their numerous advantages, concerns regarding the safety and toxicity of nanomaterials in ophthalmology have also arisen [330] **(**Fig. 14**)**. The size and shape of nanomaterials may influence their interaction with ocular tissues, potentially leading to unforeseen consequences. Small particles can easily penetrate ocular barriers and reach sensitive tissues, while specific shapes may affect their cellular uptake and biodistribution [258]. Furthermore, factors such as surface charge, hydrophobicity, and the presence of functional groups on nanomaterials can significantly impact their biocompatibility and interaction with ocular tissues. Surface modification techniques may be employed to enhance biocompatibility and reduce potential toxicity [331–333]. Additionally, the tendency of certain nanomaterials to aggregate in physiological environments can induce complications, such as occlusion of ocular blood vessels and inflammatory responses [334]. Moreover, the rate at which nanomaterials degrade and are eliminated from the body plays a crucial role in determining their potential toxicity. Substances that break down too quickly may release harmful byproducts, while those that degrade slowly may accumulate in tissues, causing prolonged damage [335]. To harness the full potential of nanomaterials in ophthalmology while mitigating their associated risks, several approaches can be adopted to minimize their toxicity: (1) Optimizing size, shape, and surface properties: By carefully selecting the dimensions, structure, and surface characteristics of nanomaterials, researchers can reduce potential adverse effects on ocular tissues. For instance, smaller particles with a more neutral surface charge are less likely to cause irritation and inflammation [336–338]; (2) Biodegradable materials: Choosing biodegradable substances that can be safely metabolized and removed from the body can help minimize the risk of lasting toxicity and tissue accumulation [339]. (3) Surface modification: Surface modification techniques, such as the addition of hydrophilic polymers, can be used to improve the biocompatibility of nanomaterials and decrease their tendency for aggregation and occlusion [340]. (4) Targeted delivery: Developing targeted drug delivery systems that can specifically deliver nanomaterials to the desired site of action within the eye can help reduce off-target effects and minimize potential toxicity [317]. (5) Preclinical assessment: Thorough preclinical evaluation of nanomaterials in relevant animal models and in vitro assays is essential to identify and address potential safety concerns before progressing to clinical trials [341].

In summary, nanomaterials have the potential to transform the field of ophthalmology, offering innovative solutions for the treatment and diagnosis of various ocular conditions. However, concerns regarding their safety and toxicity must be thoroughly addressed to ensure the successful translation of these technologies to clinical applications. By implementing strategies to minimize toxicity, optimizing material properties, and conducting rigorous preclinical testing, researchers can pave the way for the safe and effective use of nanomaterials in ophthalmology.

## Conclusion and perspective

In conclusion, the emergence of next-generation nanomaterials holds the potential to substantially advance the domain of ocular anti-inflammatory drug therapy. These groundbreaking materials present unparalleled prospects for devising novel drug delivery systems and augmenting the efficacy of existing treatments, ultimately ameliorating patient outcomes for a wide array of ocular conditions. By exploiting the unique attributes of nanomaterials, researchers can tackle challenges linked with conventional therapies, such as limited bioavailability, off-target effects, and the necessity for frequent administration.

As we gaze into the future, the sustained development and refinement of nanomaterials for ocular drug delivery will likely result in breakthroughs in the management of inflammatory eye diseases. Investigators will strive to optimize the physicochemical properties of nanomaterials, including size, shape, surface charge, and biodegradability, to attain superior biocompatibility, targeted delivery, and controlled drug release. Moreover, the incorporation of stimuli-responsive mechanisms and multifunctional capabilities may lay the foundation for intelligent, personalized therapies tailored to individual patient requirements.

Furthermore, interdisciplinary cooperation among material scientists, ophthalmologists, and pharmaceutical researchers will be pivotal in propelling the translation of these avant-garde technologies from the laboratory to the clinical setting. Rigorous preclinical evaluation and meticulously designed clinical trials will be essential in verifying the safety and efficacy of nanomaterial-based ocular therapies, ensuring compliance with regulatory standards and garnering acceptance within the medical community.

However, concomitant with the substantial promise of nanomaterials in ocular anti-inflammatory drug therapy, it is vital to remain attentive to potential safety concerns and adverse effects. Researchers must assiduously appraise the biocompatibility and toxicity of these materials, implementing strategies to minimize potential hazards while maximizing therapeutic advantages. In doing so, the full potential of next-generation nanomaterials can be harnessed to transform the landscape of ocular drug therapy, providing improved treatment options and ultimately enhancing the quality of life for millions of patients globally.

## Data Availability

Not applicable.

## Abbreviations

ON: Optic neuritis
NSAIDs: Nonsteroidal anti-inflammatory drugs
NPs: Nanoparticles
AuNPs: Gold nanoparticles
OCT: Optical coherence tomography
DEX: Dexamethasone-sodium phosphate
HA: Hyaluronic acid
LBPS: *Lycium barbarum* polysaccharides
HCFs: Human corneal fibroblasts
AMD: Age-related macular degeneration
APS: *Astragalus* polysaccharides
ARPE-19: Human retinal pigment epithelial cells
ER: Endoplasmic reticulum
DR: Diabetic retinopathy
PGBL: *Ginkgo biloba* leaf-derived polysaccharides
TNF-α: Tumor necrosis factor-α
DCPS: *Dendrobium candidum* polysaccharides
HCEC: Human corneal epithelial cells
SA: Sodium alginate
AOS: Alginate oligosaccharides
HCS: High molecular weight chitosan
SLNs: Solid lipid nanoparticles
NLCs: Nanostructured lipid carriers
LDCs: Lipid-drug conjugates
PLGA: Polylactic-co-glycolic acid
PGA: Polyglycolic acid
PEG: Polyethylene glycol
RES: Reticuloendothelial system
ASIV-LNCs: Astragaloside-IV loaded into lipid nanocapsules
PAMAM: Polyamidoamine
PLL: Poly(L-lysine)
PEI: Polyethylenimine
PPI: Poly (propylene imine)
OIR: Oxygen-induced retinopathy
TA: Triamcinolone acetonide
D-TA: Dendrimer-conjugated TA
SPIONs: Superparamagnetic iron oxide nanoparticles
PVA: Polyvinyl alcohol
MSCs: Mesenchymal stem cells
DCIONs: Dextran-coated iron oxide nanoparticles
TGF-γ: Tumor growth factor-γ
CsA: Cyclosporine A
OSI: Ocular surface inflammation
Th17: T helper cell 17
CMVR: Cytomegalovirus retinitis
MBL: Mannose-binding lectin
FFA: Fluorescein fundus angiography
VEGF: Anti-vascular endothelial growth factor
PDR: Proliferative diabetic retinopathy
RPE: Retinal pigment epithelium
MS-ON: Multiple sclerosis-related optic neuritis
NMO-ON: Neuromyelitis optica-related optic neuritis
AQP4-IgG: Antibodies against astrocyte water channel protein 4
SLE: Systemic lupus erythematosus
GMs: Graphene materials
EFMs: Electrospun fibrous membranes
ROS: Reactive oxygen species
SOD: Superoxide dismutase
CNTs: Carbon nanotubes
FET: Field-effect transistor
PCL-PEG-PCL: Polycaprolactone-polyethylene glycol-polycaprolactone
DFAB: Difluprednate
ACs: Anterior chamber cells
VRC: Voriconazole
VE: Vitamin E
PVP: Poly(vinylpyrrolidone)
PCL: Poly(ε-caprolactone)
OFX: Ofloxacin
COX: Cyclooxygenase
CS: Chitosan
HET-CAM: Hen’s egg test-chorioallantoic membrane
FB: Flurbiprofen
SA: Sodium arachidonate
HENC: Human corneal endothelial cells
HCC: Human cornea construct
LX: Lornoxicam
PBA: Phenylboronic acid
INS: In-situ nanosuspension
MS: Micellar solution
MNFs: Nanomicellar formulations
Oc-40: Octoxynol-40
NFAT: Nuclear factor of activated T cells
CD4: Differentiation cluster 4
CD8: Differentiation cluster 8
TAC: Tacrolimus
TGNPs: Tacrolimus gellan gum nanoparticles
MSNAPTES: 3-aminopropyltriethoxysilane
ERG: Electroretinography
AKC: Atopic keratoconjunctivitis
VKC: Vernal keratoconjunctivitis
NCs: Nano-capsules
EAU: Experimental autoimmune uveitis
KO: Knockout
EDE: Experimental dry eye
Myr: Myricetin
CUR: Curcumin
OA: Oleanolic acid
UA: Ursolic acid
PLA2: Phospholipase A2
SM: Standard mixture
SAS: SA solution
SDF-1: Stromal-derived factor-1
THY: Thymol
DG: Dipotassium glycyrrhizinate
ETP: Etoposide
TRAM2: Translocation-associated Membrane Protein 2
NPMOF: Nanoscale zirconium-porphyrin metal-organic frameworks
MPS: Methylprednisolone
PAM: Photoacoustic microscopy
PT-OCT: Photothermal OCT
CLs: Contact lenses
TiO_2_ NPs: Titanium dioxide nanoparticles
PMMA: Polymethyl methacrylate
SPOTS: Semiquantitative preclinical ocular toxicology scoring
LECs: Lens epithelial cells
BRB: Blood-retinal barrier
FA: Fluocinolone acetonide
LE: Loteprednol etabonate
BCEC: Bovine conjunctival epithelial cell
BCoEC: Bovine corneal epithelial cells
LPS: Lipopolysaccharide
T-HCl: Terbinafine hydrochloride
BSF: Besifloxacin hydrochloride
PC: Phosphatidylcholine
CH: Cholesterol
SA: Stearylamine
HA-LCS-NPs: Hyaluronic-acid-modified lipid-polymer hybrid nanoparticles
MRT: Mean residence time
AUC: Area under the curve
P_app_: Apparent permeability coefficients
FD-C: Franz diffusion cell
PMNLs: Polymorphonuclear leukocyte infiltration
NP-NLC-Gel: Hydrogel modified nanostructured lipid carrier
RPCECs: Rabbit corneal epithelial cells
CH-MEs: Chitosan-coated cationic microemulsions
NIPAAM: N-isopropylacrylamide
VP: Vinyl pyrrolidone
MAA: Methacrylate
P40S: Polyoxyl 40 stearate
P80: Polysorbate 80
HA CS-NPs: Hyaluronic-acid chitosan-sodium tripolyphosphate nanoparticle
Gamma CD: Gamma-cyclodextrin
Dex-SA-FFFE: Dexamethasone-peptide conjugate
HPMC: Hydroxypropyl methylcellulose
HP-β-CD: Hydroxypropyl-β-cyclodextrin
F6: Phosphatidylcholine, cholesterol, span 60, and stearylamine at a molar ratio of 1:1:1:0.15
PBA-CS-VE: Phenylboronic acid conjugated chitosan oligosaccharide-vitamin E copolymer
COL: Chitosan oligosaccharide lactate
CTAB: Hexadecyltrimethylammonium bromide
CNLC: Cationic nanostructured lipid carriers
COS: Chitosan oli gosaccharides
mPEG-b-PLGA: Methoxy poly (ethylene glycol)-block-poly (D, L)-lactic-co-glycolic acid
PVCL-PVA-PEG: Polyvinyl caprolactam-polyvinyl acetate-polyethylene glycol
GA: Gambogic acid
Vit E TPGS: Vitamin E tocopherol polyethylene glycol succinate
Oc-40: Octoxynol-40
TA: Sodium taurocholate
MBA: N, N-methylenebis-acrylamide
TG: Triethyleneglycol dimethacrylate
MC: Methylcellulose
ALG: Alginate
